# IGFBP5 alleviates periodontitis by reversing human dental follicle stem cell senescence via the non-canonical Wnt pathway

**DOI:** 10.1038/s41368-026-00463-2

**Published:** 2026-09-15

**Authors:** Yuru Wang, Yue Wang, Yuguo Dai, Weihua Guo, Jun Liu

**Affiliations:** 1 State Key Laboratory of Oral Diseases & National Center for Stomatology & National Clinical Research Center for Oral Diseases, West China Hospital of Stomatology, Sichuan University, Chengdu, China; 2 Department of Orthodontics, West China Hospital of Stomatology, Sichuan University, Chengdu, China; 3 Division of Restorative Dental Sciences, Faculty of Dentistry, The University of Hong Kong, Hong Kong SAR, China; 4 Yunnan Key Laboratory of Stomatology & Department of Pediatric Dentistry, The Affiliated Stomatology Hospital, Kunming Medical University, Kunming, China

**Keywords:** Mesenchymal stem cells, Periodontitis, Stem-cell research

## Abstract

The improvement of health and the delay of aging represent pivotal goals in medical research. Bone aging contributes to various age-related disorders, including periodontitis. Dental follicle stem cells (DFSCs) serve as ideal seed cells for periodontal regenerative therapy. Insulin-like growth factor binding protein 5 (IGFBP5) has been reported to exert complex and context-dependent effects. However, its role in DFSC senescence remains undefined. In this study, we observed a significant decrease in IGFBP5 expression in DFSCs undergoing replicative or H₂O₂-induced senescence. Functional experiments demonstrated that IGFBP5 overexpression alleviated cellular senescence and enhanced osteogenic potential, a process linked to the modulation of non-canonical Wnt signaling. WNT5B was then identified as a potential downstream regulator involved in mediating the pro-osteogenic effects of IGFBP5. To translate these findings clinically, we developed Gel-vHA@oe-DFSC, a nanoparticle-embedded hydrogel system for cell delivery, which enhanced the therapeutic efficacy of DFSCs and promoted periodontal regeneration in a rat model. Collectively, our study reveals a novel function of IGFBP5 in mitigating DFSC senescence and presents a promising strategy for combating periodontal bone loss.

## Introduction

Aging is a comprehensive process marked by a decline in physiological functions across tissues and organs.^1^ Bone aging, characterized by reduced bone mass and density, is an early manifestation of aging.^2^ The balance of bone remodeling, orchestrated by osteoblasts and osteoclasts, is disrupted in age-related bone diseases, leading to bone loss.^3^ Periodontitis, one of the age-related bone diseases, primarily targets the tooth-supporting tissues with chronic inflammation, and, in severe cases, it can lead to aggressive and irreversible destruction of periodontal structures.^4^ While current treatments often rely on invasive surgery, stem cell therapy offers a promising alternative.^5^ Implanted stem cells not only serve as units for regeneration, but also enhance resident cell activity through signaling molecules, rebalancing the local environment.^6,7^ A recent multicenter clinical trial in China demonstrated that allogeneic dental stem cell injections improve clinical outcomes in periodontitis, highlighting the potential of this minimally invasive strategy.^8^

Dental stem cells (DSCs), neural crest-derived mesenchymal stem cell (MSC)-like populations, exhibit self-renewal, multipotency and low immunogenicity.^9^ Among them, dental follicle stem cells (DFSCs) are particularly promising due to their accessibility, primitive state, high proliferation, and strong multi-lineage differentiation potential, making them ideal for oral regenerative therapy.^10,11^ Even after periodontal tissue maturation, DFSCs still play essential roles in maintaining periodontal tissue homeostasis by supplementing aging functional cells and mediating repair after injury.^12^ Stem cell senescence, however, impairs tissue maintenance and repair, and therapeutic cells are similarly susceptible.^13^ Replicative arrest in aged or long-term cultured cells limits their therapeutic utility.^14^ In periodontitis, senescence of seed cells can exacerbate local inflammation and disrupt bone remodeling, creating a self-perpetuating cycle that contributes to treatment failure.^15^ Although DSC senescence is known to impair stemness and osteogenesis, its regulatory mechanisms remain incompletely understood.

Insulin-like growth factor binding protein 5 (IGFBP5), the most conserved member of the insulin-like growth factor (IGF)-binding protein (IGFBP) family, is a secretory protein that binds IGF with high affinity, influencing cell survival and growth via insulin-like growth factor 1 receptor (IGF1R) signaling.^16^ Beyond IGF-dependent roles, IGFBP5 also exerts IGF-independent functions, including transcriptional regulation and modulation of tumor growth.^17^ Its effects vary across cell types and pathways, necessitating mechanistic studies.^18^ Notably, IGFBP5 is highly expressed in bone and promotes osteogenic differentiation of MSCs.^19,20^ It enhances proliferation and osteogenesis in periodontal ligament stem cells and is downregulated in periodontitis models, while recombinant IGFBP5 promotes periodontal repair via JNK/ERK signaling.^21,22^ However, whether IGFBP5 alleviates senescence and regulates osteogenesis in DFSCs, critical for their application in aging-related periodontal therapy, remains unknown.

Direct administration presents additional challenges for the survival and integration of DFSCs into target tissues.^23^ To enhance cell viability, the use of biocompatible scaffolds such as Gelatin methacryloyl (GelMA) hydrogels has been proposed.^24^ GelMA represents an ideal carrier for the topical delivery of MSCs in cell therapy and is also commonly employed as a composite scaffold, incorporated with nanohydroxyapatite (nHA) and polymers to mimic the natural composition of periodontium.^25^ Previous studies have shown that vinyl-modified nanohydroxyapatite (vHA) improves dispersion within composite matrices through tailored surface functionalization, which optimizes its structural integration and ultimately enhances bone regeneration.^26^ Nevertheless, the effect of GelMA loaded with genetically modified DFSCs and interface-integrated nHA on alleviating cellular senescence and enhancing bone regeneration has not yet been determined.

This study sought to investigate key molecular drivers of DFSC aging and their therapeutic potential. First, gene expression profiling identified IGFBP5 as being significantly downregulated in aged DFSCs. We then demonstrated that overexpressing IGFBP5 alleviated cellular senescence and enhanced osteogenic differentiation through the non-canonical Wnt pathways. Our work sought to definitively establish the functional impact of IGFBP5 through comprehensive in vitro and in vivo experiments, with the ultimate goal of evaluating IGFBP5-overexpressing DFSCs as a stem cell-based therapy for periodontitis, thereby proposing a new treatment for age-related bone loss.

## Results

### IGFBP5 expression declines during DFSC senescence

**Fig. 1:**
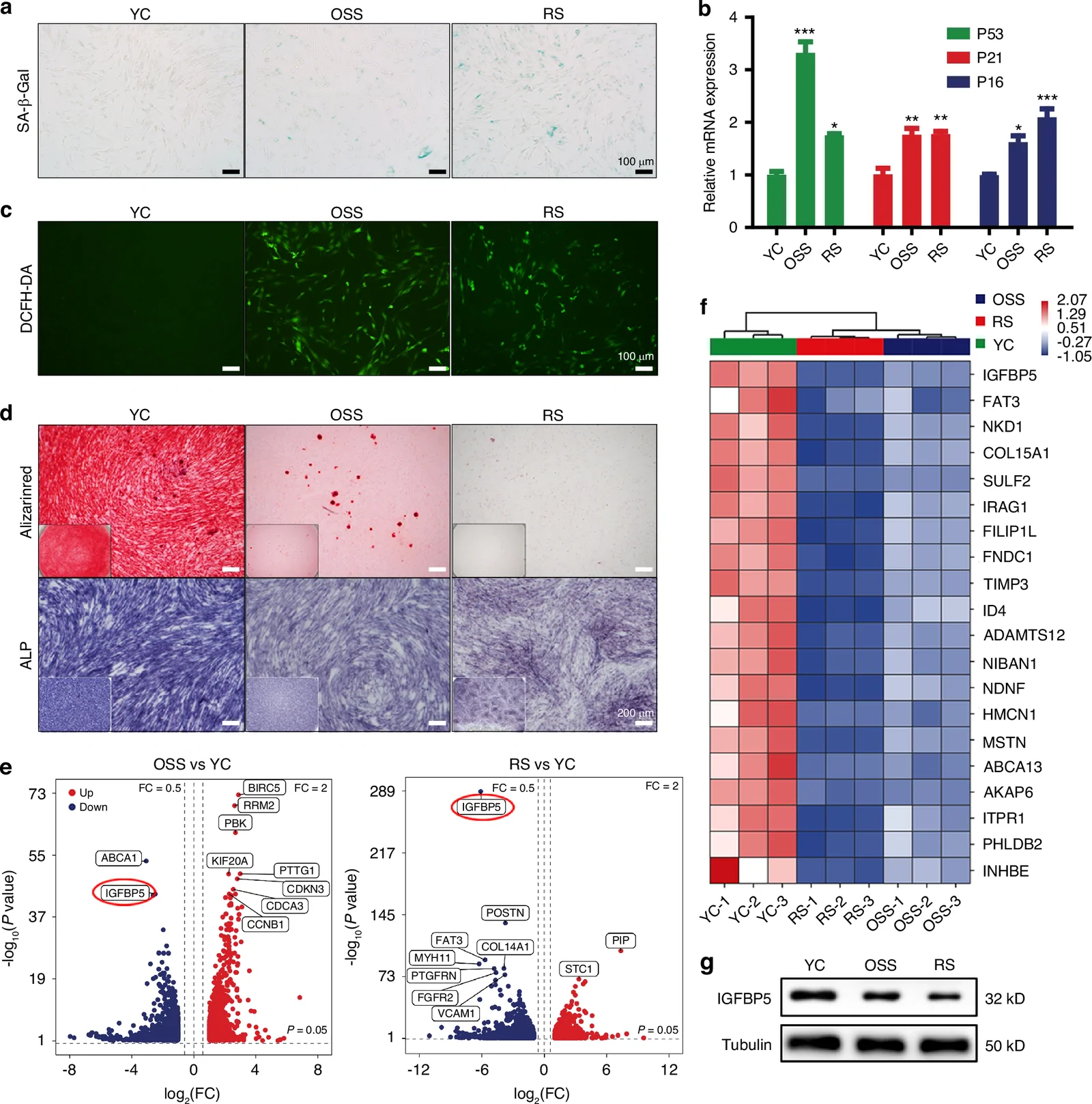
IGFBP5 expression declines during DFSC senescence. **a** SA-β-gal staining to detect cell senescence (scale bar: 100 μm). **b** qRT-PCR analysis of the expression of P53, P21, and P16 mRNA. **c** DCFH-DA staining to detect intracellular ROS (scale bar: 100 μm). **d** Alizarin red and ALP staining to detect osteogenic differentiation (scale bar: 200 μm). **e** Volcano plots showing DEGs in OSS vs YC and RS vs YC. Dotted vertical lines represent fold change = 2 (right, up-regulation) and fold change = 0.5 (left, down-regulation). **f** Heatmap of representative expression for 20 downregulated genes in senescent groups relative to YC. **g** Western blot assay showing the expression of IGFBP5. Data are presented as the mean ± SD (*n* = 3). *P < 0.05; **P < 0.01; ***P < 0.001

DFSCs were characterized and met international standards.^27^ Senescence was induced via H₂O₂ treatment (oxidative stress-induced senescence, OSS) or serial passaging (replicative senescence, RS). Increased senescence-associated β-galactosidase (SA-β-gal) staining was observed in both models (Fig. 1a) and elevated expression of cell cycle arrest genes (P53, P21, P16) (Fig. 1b), confirming successful senescence induction. We also observed enhanced DCFH-DA fluorescence signal, suggesting elevated intracellular reactive oxygen species (ROS) levels (Fig. 1c), alongside reduced cell migration, viability (Supplementary Fig. S1a, b), ALP activity, and mineralization (Fig. 1d). Expression of osteogenic markers (ALP, RUNX2, OCN) was also diminished (Supplementary Fig. S1c).

RNA sequencing (RNA-seq) of young cells (YC), OSS, and RS groups revealed that senescence accounted for most variances, with tight clustering of biological replicates confirming data reliability (Supplementary Fig. S2a). Differential expression analysis identified 1978 differentially expressed genes (DEGs) (1034 upregulated and 944 downregulated) in OSS and 2764 DEGs (1278 upregulated, 1486 downregulated) in RS versus YC (Supplementary Fig. S2b). IGFBP5 emerged as highly senescence-sensitive, downregulated >5-fold in OSS and 69-fold in RS models (Fig. 1e, f). Gene Ontology (GO) analysis indicated that downregulated DEGs in both models were enriched in system development and extracellular matrix organization (Supplementary Fig. S2c, d). qRT-PCR and western blot confirmed IGFBP5 downregulation at both mRNA and protein levels (Fig. 1g, and Supplementary Fig. S2e). These findings demonstrate that DFSC senescence impairs osteogenesis, with IGFBP5 as a potential key regulator.

### IGFBP5 overexpression protects DFSCs against H_2_O_2_-induced senescence

We constructed sh-IGFBP5 and oe-IGFBP5 lentiviral vectors to modulate IGFBP5 expression (Supplementary Fig. S3a). Knockdown efficiency was confirmed at mRNA and protein levels (Supplementary Fig. S3b, c). IGFBP5 knockdown increased SA-β-gal-positive cells (Supplementary Fig. S4a), elevated P21 and P16 levels (Supplementary Fig. S4b), increased ROS, and decreased cell viability (Supplementary Fig. S4c, d). Osteogenic potential was also impaired, with downregulated ALP, RUNX2, OCN expression and reduced mineralization (Supplementary Fig. S4b, e).

**Fig. 2:**
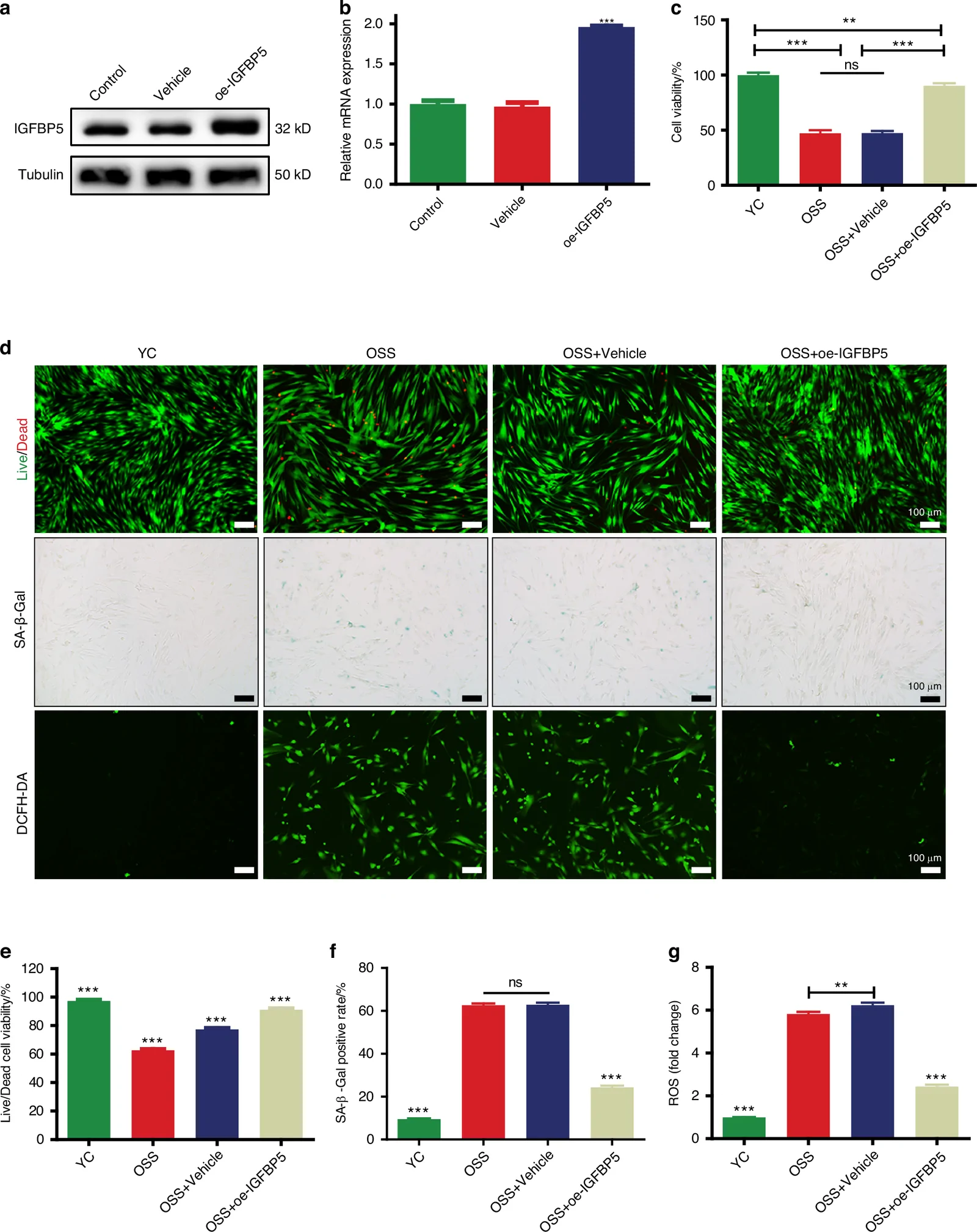
IGFBP5 overexpression alleviates H_2_O_2_-induced cytotoxicity, senescence, and oxidative stress in DFSCs. **a**, **b** Western blot assay and qRT-PCR analysis showing the expression of IGFBP5 to verify overexpression of IGFBP5 compared with the vehicle control group. **c** CCK-8 assay to determine cell viability. **d** Live/dead staining to assess cell viability, SA-β-gal staining to detect cell senescence and DCFH-DA staining to detect intracellular ROS (scale bar: 100 μm). **e** Quantification of the percentage of live cells. **f** Quantification of the percentage of senescent SA-β-gal-positive cells. **g** Quantification of fluorescence intensity. Data are presented as the mean ± SD (*n* = 3). ns, no significance; **P < 0.01; ***P < 0.001

**Fig. 3:**
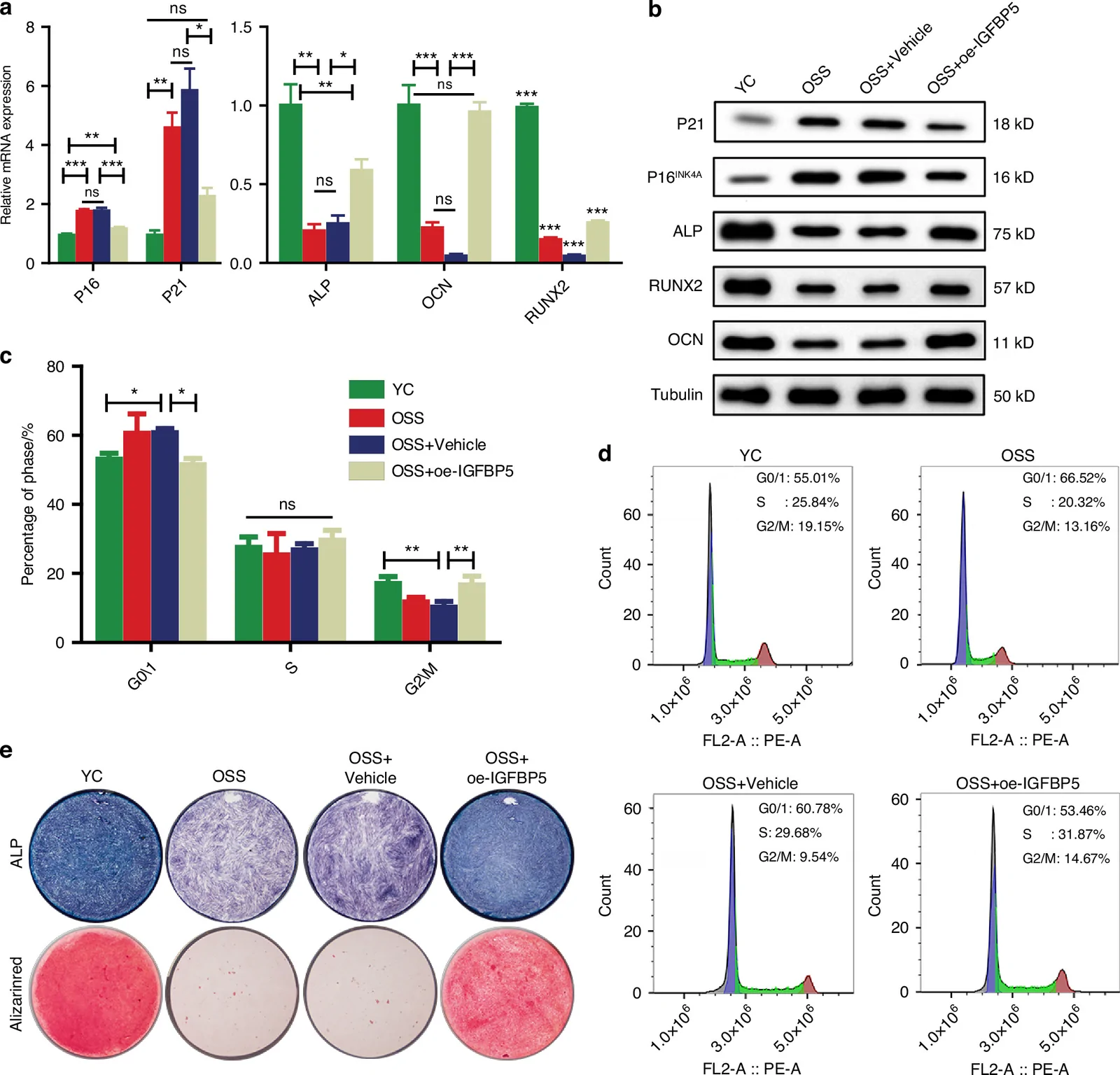
IGFBP5 overexpression reverses H_2_O_2_-induced cell cycle arrest and preserves osteogenic differentiation potential of DFSCs. **a** The mRNA levels of aging-related genes (P16 and P21) and osteogenic-related genes (ALP, OCN and RUNX2). **b** Western blot of P21, P16^INK4A^, ALP, RUNX2 and OCN protein levels. **c** The percentage of DFSCs in G0/G1, S, and G2/M phases of cell cycle. **d** PI staining coupled with flow cytometry to analyze cell cycle distribution. **e** ALP staining for early osteogenic differentiation and alizarin red staining for calcium deposition to assess osteogenic potential. Data are presented as the mean ± SD (*n* = 3). ns, no significance; *P < 0.05; **P < 0.01; ***P < 0.001

We next overexpressed IGFBP5 to reverse its senescence-associated decline. Successful overexpression was confirmed by qRT-PCR and western blot (Fig. 2a, b). IGFBP5 overexpression rescued H₂O₂-induced loss of cell viability (Fig. 2c) and increased the proportion of live cells (Fig. 2d, e). It also reduced key senescence markers: SA-β-gal-positive cells (Fig. 2d, f), P21 and P16 expression (Fig. 3a, b), and intracellular ROS levels (Fig. 2d, g). Cell cycle analysis showed that IGFBP5 alleviated H₂O₂-induced G0/G1 arrest (Fig. 3c, d). Osteogenic potential, compromised by senescence, was restored, as shown by upregulated osteogenic markers and enhanced mineralization (Fig. 3a, b, e). Notably, except for OCN, which showed no significant change, RUNX2 and ALP expression in the OSS+oe-IGFBP5 group remained lower than in YC (Fig. 3a). These results demonstrate that IGFBP5 overexpression protects against H₂O₂-induced senescence in DFSCs.

### IGFBP5 attenuates DFSC senescence via the non-canonical Wnt pathway

**Fig. 4:**
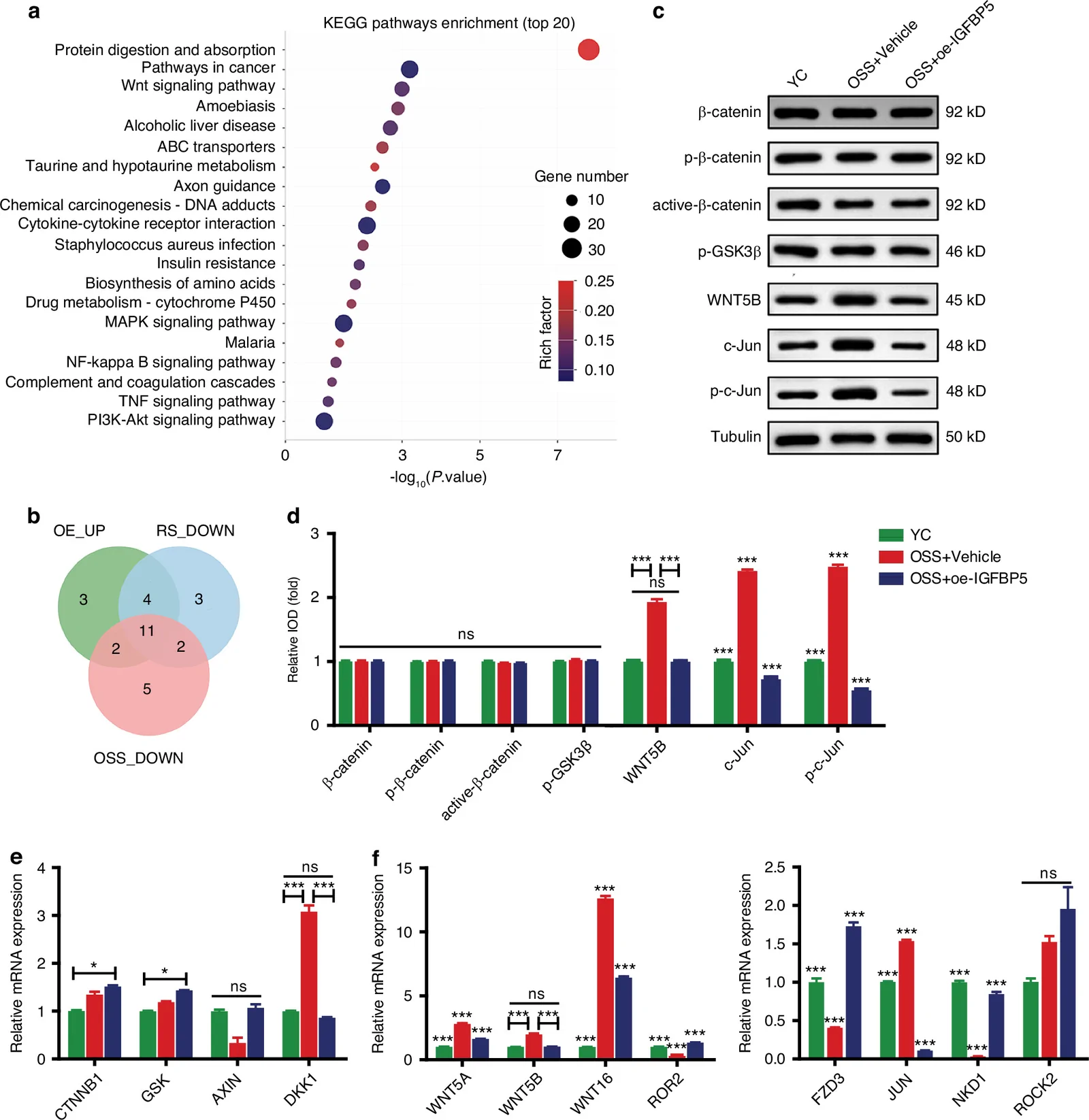
Overexpression of IGFBP5 regulates the non-canonical Wnt pathway. **a** KEGG enrichment analysis of the top 20 enriched pathways, with the Wnt signaling pathway ranked among the top three. **b** Venn diagram showing intersection of KEGG enrichment downregulated in OSS and RS compared with YC, yet upregulated in the OSS+oe-IGFBP5 group compared with OSS+Vehicle group. **c**, **d** Western blot analysis and quantification of the expression of β-catenin, phospho-β-catenin, active-β-catenin, phospho-GSK3β, WNT5B, c-Jun, and phospho-c-Jun proteins, and quantification of these protein expressions. **e** qRT-PCR analysis of the expression of mRNA related to the canonical Wnt/β-catenin pathway including CTNNB1, GSK, AXIN and DKK1. **f** qRT-PCR analysis of the expression of mRNA related to the non-canonical Wnt pathways including Wnt ligands (WNT5A, WNT5B, WNT16) and related genes (ROR2, FZD3, JUN, NKD1, ROCK2). Data are presented as the mean ± SD (*n* = 3). ns, no significance; *P < 0.05; ***P < 0.001

To explore the mechanism, we performed RNA-seq on OSS+oe-IGFBP5 and OSS+Vehicle groups. We identified 796 upregulated and 582 downregulated DEGs, including upregulated osteogenesis-related genes like collagens and ALP (Supplementary Figs. S5a and S6a). GO analysis revealed enrichment in “developmental process”, “morphogenesis”, and “extracellular matrix” terms (Supplementary Fig. S6b). Kyoto Encyclopedia of Genes and Genomes (KEGG) analysis indicated significant regulation of the Wnt signaling pathway, which was similarly enriched in the aging-related contrasts (Fig. 4a, and Supplementary Fig. S7). Comparing upregulated terms in OSS+oe-IGFBP5 vs. OSS+Vehicle with downregulated terms in senescence models identified 11 consistently altered pathways (Fig. 4b).

The Wnt/β-catenin, Wnt-planar cell polarity (PCP), and Wnt-Ca²⁺ pathways represent three well-characterized branches of Wnt signaling that share certain molecular components yet exert distinct roles during osteogenic differentiation of stem cells.^28^ Genomic data showed pronounced expression changes in Wnt signaling components, particularly those associated with non-canonical pathways (Supplementary Fig. S5b–h). Several non-canonical Wnt ligands (WNT5B, WNT10B, WNT16) and related genes (ROR2, NKD1, DAAM2) were differentially expressed. Western blot analysis showed that IGFBP5 overexpression markedly decreased protein levels of non-canonical pathway markers (WNT5B, c-Jun, phospho-c-Jun), while canonical pathway components (β-catenin, phospho-β-catenin, phospho-GSK3β) remained unchanged (Fig. 4c, d). Consistent with this, qRT-PCR revealed relatively modest changes in core canonical Wnt genes (CTNNB1, GSK, AXIN), with the exception of the canonical pathway inhibitor DKK1, which showed significant mRNA-level alteration (Fig. 4e). In contrast, multiple non-canonical genes (WNT5A, WNT5B, WNT16, ROR2, FZD3, JUN, NKD1) exhibited significant differential expression (Fig. 4f). These results suggest that IGFBP5 primarily influences the non-canonical Wnt pathway in senescent DFSCs, with WNT5B as a potential key regulator.

### WNT5B acts as a downstream effector of IGFBP5 in modulating osteogenesis

**Fig. 5:**
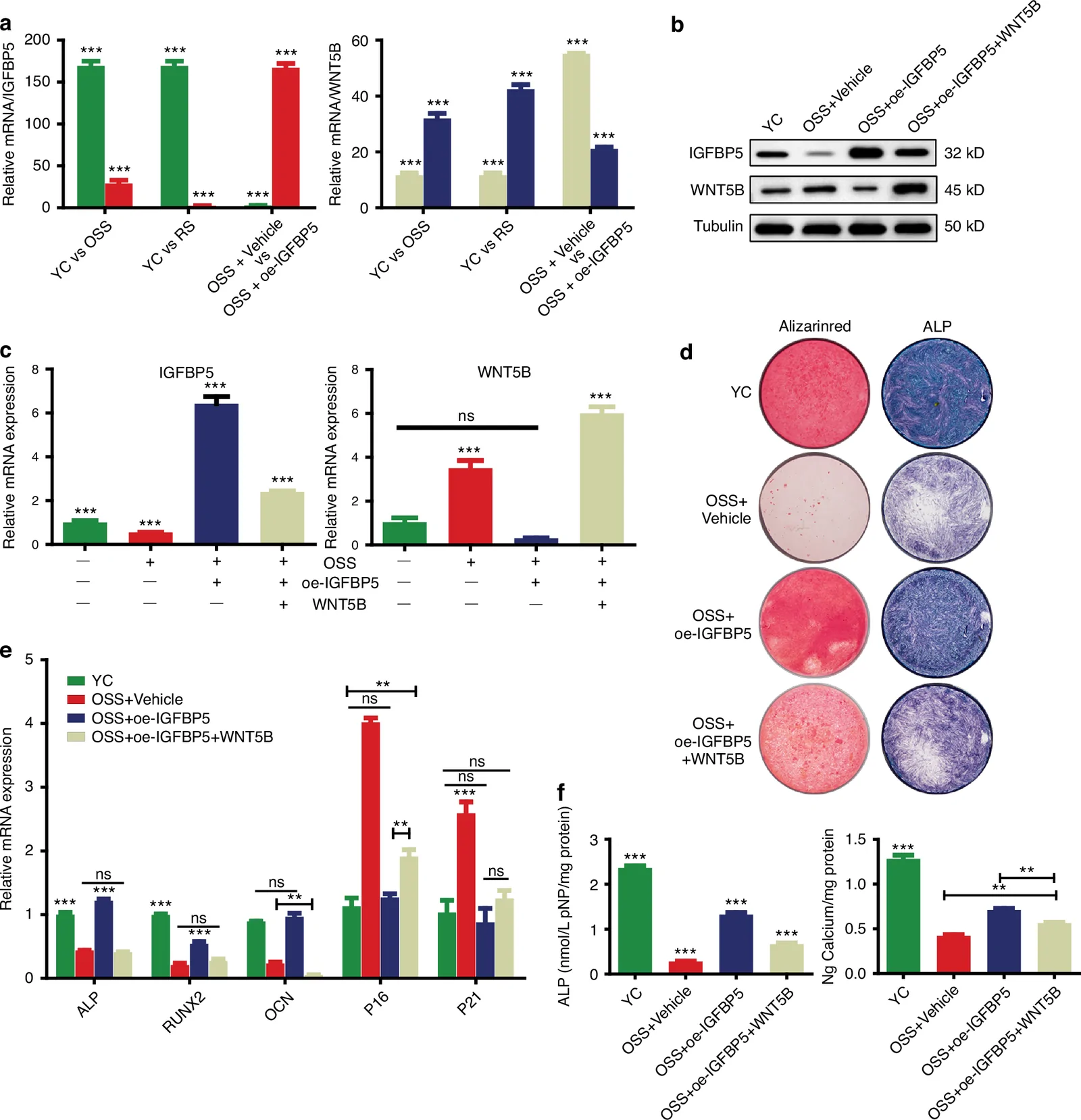
WNT5B acts as a potential downstream target of IGFBP5 in modulating osteogenesis. **a** RNA-seq analysis showing relative expression changes (fold change) of IGFBP5 and WNT5B in the indicated comparisons. **b** Western blot analysis of the expression of IGFBP5 and WNT5B proteins. **c** qRT-PCR analysis of the expression of IGFBP5 and WNT5B mRNA. **d** ALP staining to detect ALP activity and alizarin red staining to detect calcium deposition. **e** The mRNA levels of aging-related genes (P16 and P21) and osteogenic-related genes (ALP, OCN and RUNX2). **f** Quantification of ALP activity and mineralized nodules. Data are presented as the mean ± SD (*n* = 3). ns, no significance; **P < 0.01; ***P < 0.001

To investigate the relationship between IGFBP5 and WNT5B during senescence and osteogenesis, we first re‑examined our RNA‑seq data from both senescence models. We compared the expression levels of IGFBP5 and WNT5B in three relevant contrasts: YC vs. OSS, YC vs. RS, and OSS+Vehicle vs. OSS+oe‑IGFBP5 (Fig. 5a). The data clearly showed that in both OSS and RS models, senescence was associated with decreased IGFBP5 and increased WNT5B expression, while overexpression of IGFBP5 in OSS cells significantly downregulated WNT5B. This consistent inverse correlation strongly suggested that the IGFBP5/WNT5B regulatory axis is operative under aging conditions. Accordingly, we found that oe-IGFBP5 significantly downregulated WNT5B at both protein and mRNA levels (Fig. 5b, c).

WNT5B plays diverse roles in bone physiology, and its inactivation has been shown to elevate bone mineral density.^29^ To determine if IGFBP5 influences WNT5B during osteogenesis, we treated cells with recombinant human WNT5B (rhWNT5B) protein in vitro. Adding rhWNT5B decreased endogenous IGFBP5 expression, indicating a potential mutual negative regulation (Fig. 5b, c). Based on the finding that WNT5B suppresses osteoblast differentiation,^30^ we assessed its effect on osteogenic differentiation. ALP and alizarin red staining showed that oe-IGFBP5 enhanced mineralized nodule formation, an effect that was partially diminished by adding rhWNT5B (OSS+oe-IGFBP5 + WNT5B group) (Fig. 5d, f). Consistently, WNT5B addition decreased the expression of osteogenic markers (ALP, RUNX2, OCN) in IGFBP5-overexpressing DFSCs under oxidative stress (Fig. 5e). Furthermore, rhWNT5B increased P16 mRNA levels but did not significantly alter P21 in the OSS+oe-IGFBP5 + WNT5B group (Fig. 5e). These results indicated that IGFBP5 enhances osteogenic fate in DFSCs in part through suppression of WNT5B and its associated non-canonical Wnt signaling.

To further validate the physiological relevance of the IGFBP5/WNT5B axis, we extended our analysis to the RS model. Considering the challenges of low infection efficiency and poor cell viability in IGFBP5-overexpressing RS-DFSCs, we instead treated them with a WNT5B neutralizing antibody. Treatment with anti‑WNT5B significantly reduced SA‑β‑Gal positivity, downregulated the senescence markers P16, and upregulated the osteogenic markers at mRNA levels (Supplementary Fig. S8). Furthermore, osteogenic staining was markedly enhanced in the anti-WNT5B-treated group compared to the untreated RS group (Supplementary Fig. S8a). However, the change in p21 expression following anti-WNT5B treatment in RS cells was not statistically significant, and the rescue of senescence and osteogenic phenotypes remained partial compared to YC levels (Supplementary Fig. S8b). These results demonstrated that inhibiting WNT5B alone in RS cells partially reverses the senescence phenotype and restores osteogenic potential.

### A vinyl-nHA reinforced GelMA hydrogel is designed as enhanced cell scaffolds

**Fig. 6:**
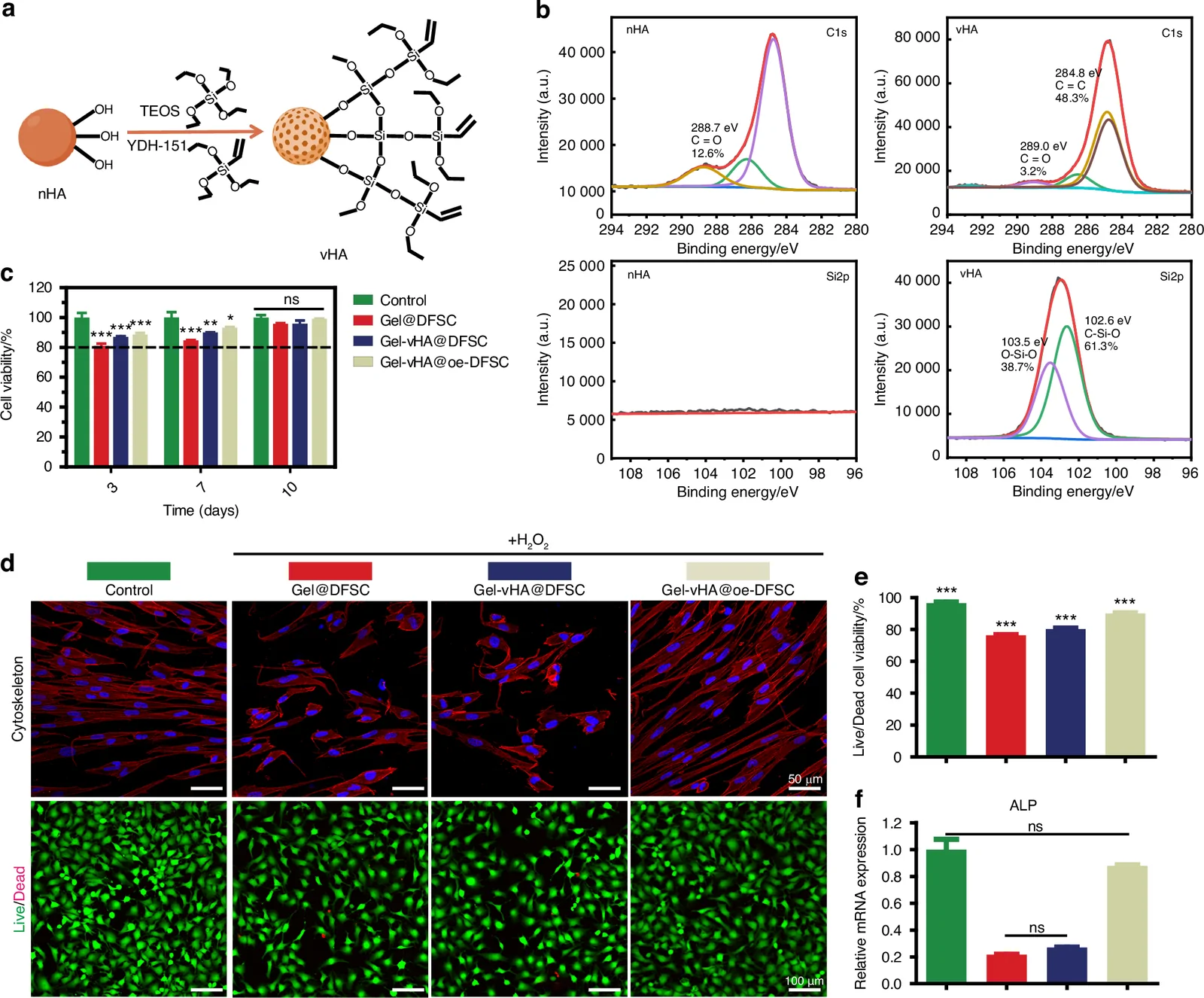
Gel-vHA@oe-DFSC demonstrates the ability to protect cell function and promote osteogenic differentiation. **a** Schematic diagram for the preparation of vHA starting from nHA precursors. **b** High-resolution XPS spectra of Si2*p* and C1*s* in nHA/vHA. **c** DFSCs viability in the indicated groups at 3, 7, and 10 days under H₂O₂-induced oxidative stress measured by CCK-8. **d** Cytoskeleton staining and live/dead staining of DFSCs-laden scaffolds at 7 days after H₂O₂ treatment (scale bar: 50 μm and 100 μm, respectively). **e** Quantification of the percentage of live cells incubated with scaffolds. **f** ALP mRNA expression of DFSCs in each group. Data are presented as the mean ± SD (*n* = 3). ns, no significance; *P < 0.05; **P < 0.01; ***P < 0.001

To standardize the application of IGFBP5-overexpressing DFSCs for periodontal treatment, we engineered a GelMA-based hydrogel to deliver modified nHA and stem cells. Vinyl-functionalized nHA was synthesized by grafting tetraethyl orthosilicate (TEOS) and triethoxyvinylsilane (silane coupling agent YDH-151) onto the nHA surface (Fig. 6a). Successful modification was confirmed by X-ray photoelectron spectroscopy (XPS), showing new Si 2*p* peaks and an enhanced C1s peak in vHA compared to pristine nHA, indicating the incorporation of silicon and vinyl groups (Fig. 6b). X-ray diffraction (XRD) confirmed the retention of nHA’s crystalline structure post-modification (Supplementary Fig. S9a), and Fourier-transform infrared (FT-IR) spectroscopy showed new vibrational peaks for C=C and C–H bonds (Supplementary Fig. S9b).

Dynamic light scattering (DLS), scanning electron microscopy (SEM), and transmission electron microscopy (TEM) indicated that vinyl modification altered nHA’s particle size and aggregation (Supplementary Fig. S9c, d). The compressive Young’s modulus of Gel-vHA was significantly higher than Gel-nHA (Supplementary Fig. S9e, f), likely due to covalent integration of vHA into the GelMA network. Rheological measurements showed stable, elastic networks in all hydrogels, with Gel-1%vHA exhibiting a significantly higher storage modulus than controls (Supplementary Fig. S9g). SEM revealed Gel-vHA formed a porous scaffold with uniform pore distribution (Supplementary Fig. S9h). Swelling experiments showed Gel-vHA had a lower swelling ratio than pure Gel (Supplementary Fig. S9i), and degradation experiments demonstrated superior weight stability for Gel-vHA initially (Supplementary Fig. S9j). Collectively, vHA integration with GelMA yielded a composite scaffold with enhanced mechanical support for cell-laden construction.

### Gel-vHA@oe-DFSC promotes osteogenic differentiation and periodontal regeneration

Integrating modified nHA improves interfacial compatibility with GelMA, enhancing osseointegration.^31^ We evaluated the effects of Gel-vHA on DFSC proliferation, differentiation, and therapeutic potential for periodontitis. Cell Counting Kit 8 (CCK-8) assays revealed that Gel-vHA restored cell viability under H₂O₂-induced oxidative stress on days 3 and 7 (Fig. 6c). Live/dead staining after 7 days of co-culture showed reduced dead cells and enhanced live cell proliferation in the H₂O₂ + Gel-vHA@oe-DFSC group, indicating favorable biocompatibility (Fig. 6d, e). Cytoskeleton staining showed increasing cell spreading area, suggesting Gel-vHA facilitates DFSC adhesion and proliferation (Fig. 6d). Moreover, ALP mRNA expression was significantly restored in the Gel-vHA@oe-DFSC group on day 14 (Fig. 6f). These results demonstrate Gel-vHA hydrogel with oe-IGFBP5 effectively promotes DFSC proliferation and osteogenic differentiation in vitro.

**Fig. 7:**
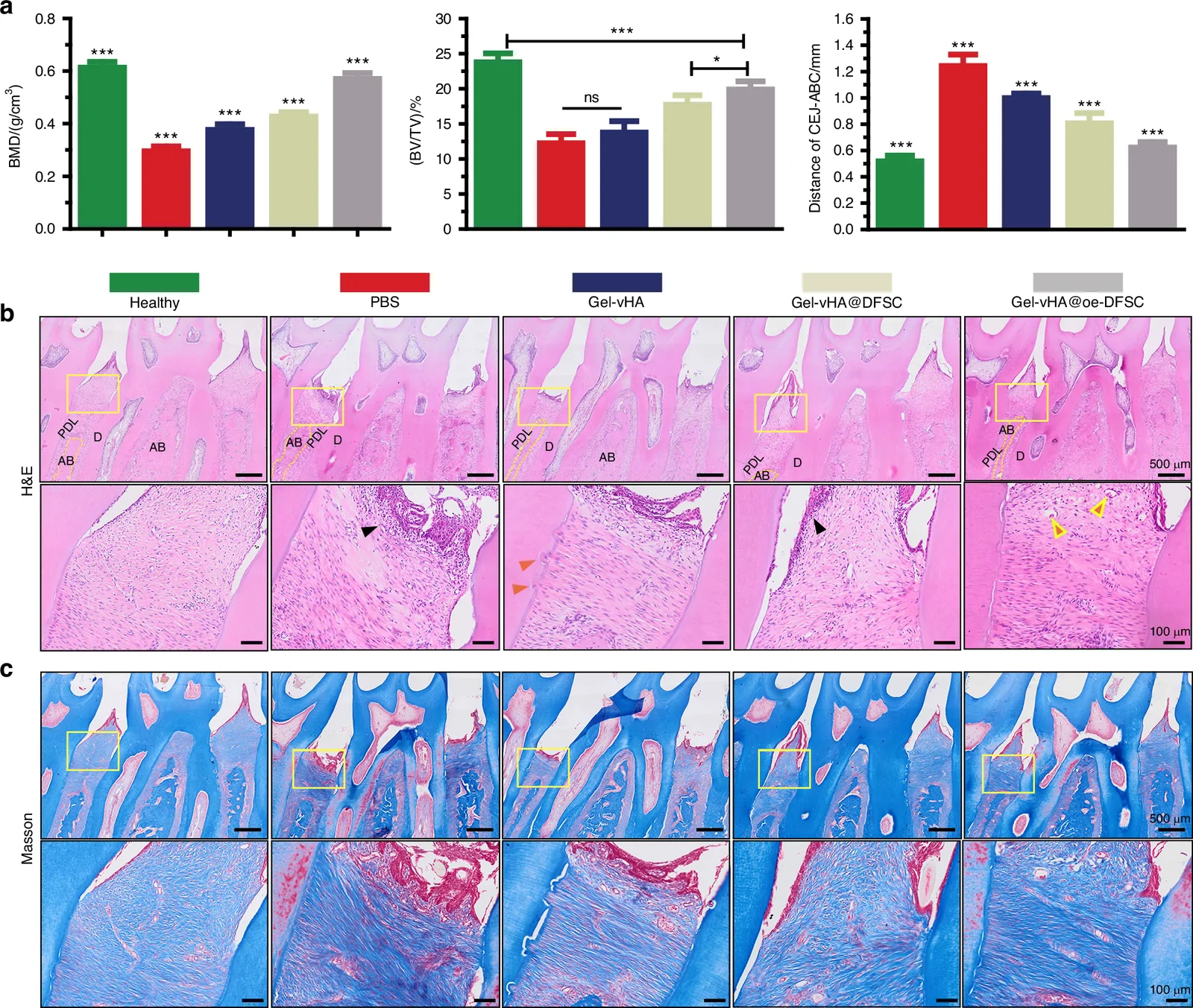
Gel-vHA@oe-DFSC promotes osteogenic differentiation and periodontal regeneration in rats. **a** Quantification of bone mineral density (BMD), bone volume fraction (BV/TV), and the distance from the cemento-enamel junction (CEJ) to the alveolar bone crest (ABC) in the indicated groups (*n* = 6). **b** Representative hematoxylin and eosin (H&E)-stained sections of periodontal tissue from each group. Yellow boxes indicate regions shown at higher magnification in the lower panel (scale bar: 500 μm; 100 μm). Black arrowheads indicate inflammatory cell infiltration; brown arrowheads indicate bone resorption lacunae (Howship lacunae); yellow open arrowheads indicate blood vessels. **c** Representative Masson’s trichrome-stained sections showing collagen deposition and tissue remodeling from each group. Collagen fibers were stained blue, whereas cytoplasm and muscle fibers were stained red (scale bar: 500 μm for upper panels and 100 μm for lower panels). Data are presented as the mean ± SD. ns, no significance; *P < 0.05; **P < 0.01; ***P < 0.001

To validate the treatment effect on tissue repair, we used a rat periodontitis model induced by ligating maxillary second molars. Rats were randomly divided into five groups: Healthy Control; PBS (untreated periodontitis); Gel-vHA (cell-free hydrogel); and two cell-therapy groups (Gel-vHA@DFSC and Gel-vHA@oe-DFSC). Bone regeneration was evaluated by micro-CT 4 weeks post treatment. Representative 3D reconstructions and 2D cross-sections are shown in Supplementary Fig. S10. The distance between the cemento-enamel junction (CEJ) and alveolar bone crest (ABC) was significantly reduced in the Gel-vHA@oe-DFSC group, being the closest among all treatments to the healthy control. Quantitative micro-CT analysis showed the Gel-vHA@oe-DFSC group exhibited superior alveolar bone quality, with higher bone mineral density (BMD) and bone volume fraction (BV/TV) (Fig. 7a).

**Fig. 8:**
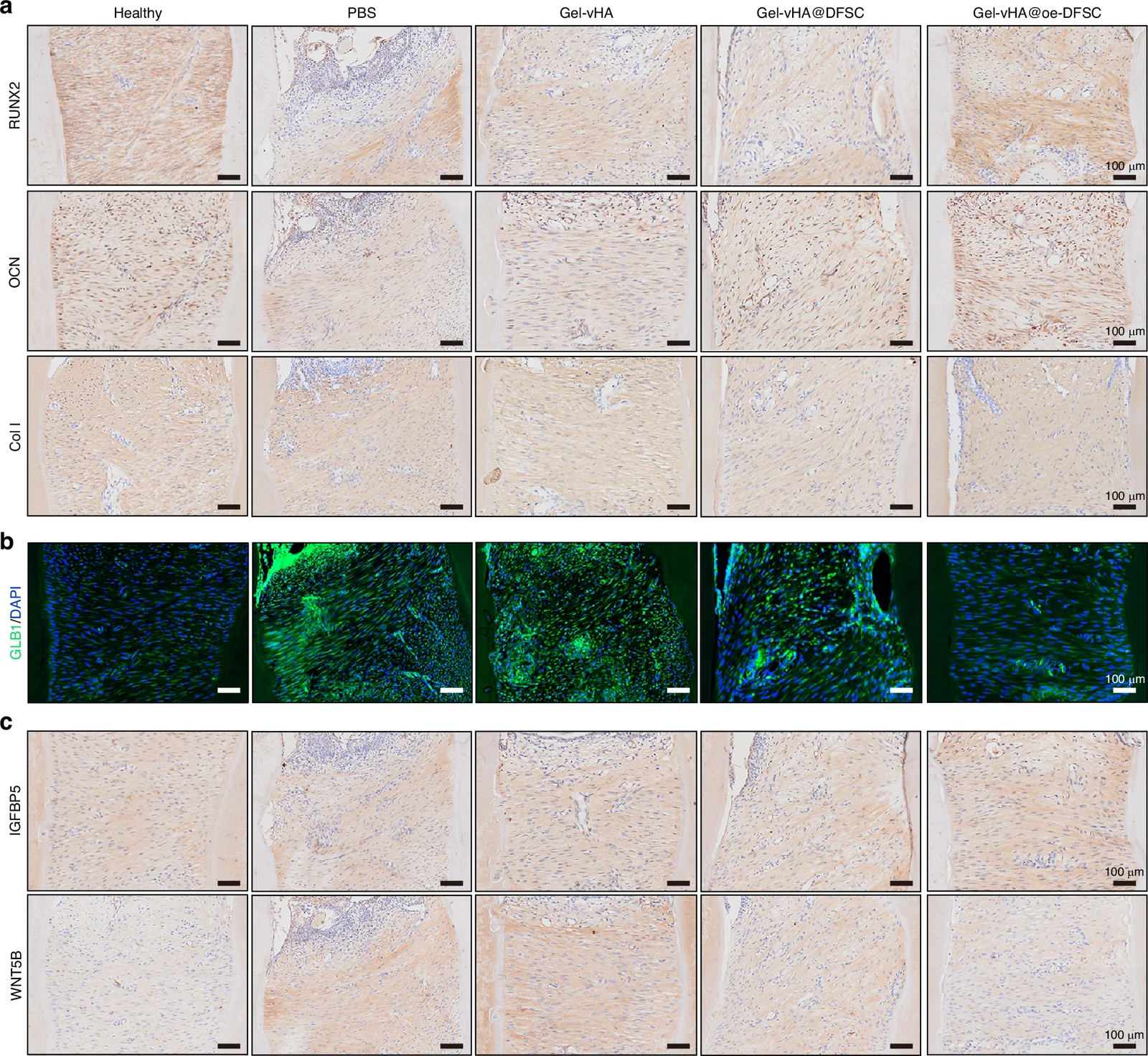
Gel-vHA@oe-DFSC upregulates osteogenic markers and modulates IGFBP5/WNT5B signaling in periodontal tissue. **a** Immunohistochemistry (IHC) staining for osteogenic markers (RUNX2, OCN, and Col I) in periodontal tissue sections. **b** Immunofluorescence (IF) of GLB1 (green) and DAPI (blue) in periodontal tissue. **c** IHC detection of IGFBP5 and WNT5B in periodontal tissue

Hematoxylin and eosin (H&E) staining of alveolar bone and periodontium showed the PBS group had impaired healing with thin epithelium, severe inflammation, and resorbed bone crest (Fig. 7b). In contrast, both cell-therapy groups could control infection and promote recovery, with Gel-vHA@oe-DFSC showing minimal inflammatory infiltration and greatest similarity to healthy tissues. Masson’s trichrome staining revealed significant tissue degeneration in the PBS group, which was notably reversed by Gel-vHA@oe-DFSC treatment, showing dense, aligned collagen bundles (Fig. 7b). Immunohistochemical (IHC) analysis revealed robust RUNX2 induction and significant Col-Ⅰ upregulation in the Gel-vHA@oe-DFSC group compared to PBS, indicating potent osteogenesis stimulation (Fig. 8a). In addition, the aging-associated protein GLB1 was upregulated in periodontitis rats and downregulated after Gel-vHA@oe-DFSC treatment (Fig. 8b). Additionally, implantation of oe-DFSC elevated local IGFBP5 levels above the PBS-treated group while reducing WNT5B, validating in vitro pathway observations (Fig. 8c). These results suggest Gel-vHA@oe-DFSC hydrogel has promising bone regeneration performance in rat periodontal defects.

## Discussion

**Fig. 9:**
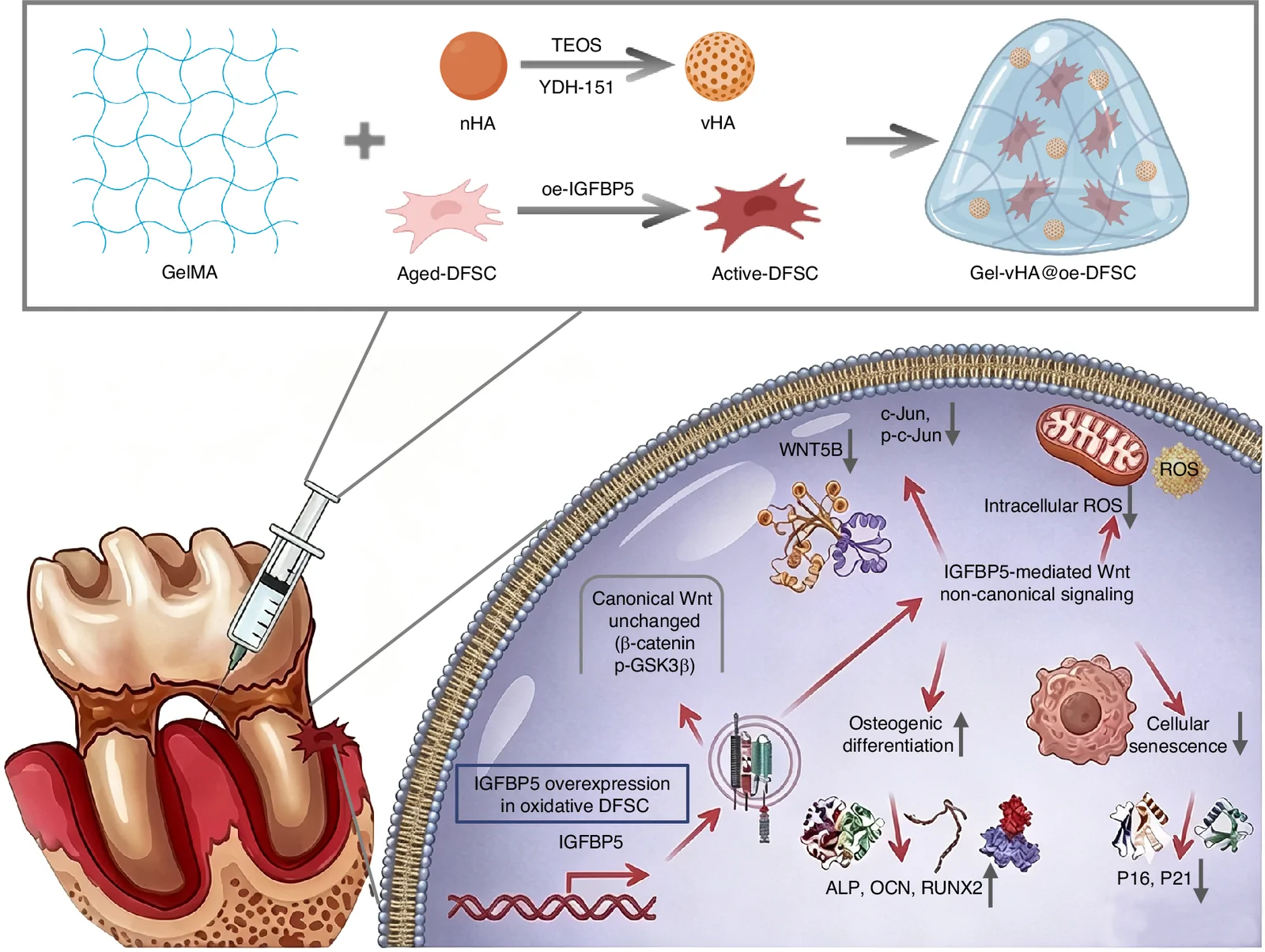
Schematic diagram showing the fabrication of Gel-vHA@oe-DFSC and its mechanism in rescuing cellular senescence and periodontal bone loss. GelMA was combined with vHA to synthesize the composite hydrogel, which was loaded with IGFBP5-overexpressing DFSCs (oe-DFSC) for in vivo delivery. Upon implantation into the periodontal defect, the hydrogel maintained cell viability under oxidative stress and supported local delivery of oe-DFSCs. Within the transplanted cells, IGFBP5 overexpression downregulated WNT5B and its downstream effector c-Jun, thereby modulating non-canonical Wnt signaling, whereas canonical Wnt/β-catenin signaling remained unaffected. These molecular changes were accompanied by attenuated cellular senescence, reduced oxidative stress, and enhanced osteogenic differentiation, collectively promoting periodontal bone regeneration

In this study, we investigated the role of IGFBP5 in DFSC senescence and its therapeutic potential for periodontitis-associated bone defects. We established the genomic profile of senescent DFSCs and confirmed that aging compromises both proliferative capacity and differentiation potential. Our data revealed a decline in IGFBP5 expression during this process and demonstrated that its restoration attenuates oxidative stress while rescuing the osteogenic program. Mechanistically, IGFBP5 suppresses non-canonical Wnt signaling by downregulating WNT5B, thereby rejuvenating cellular function and promoting bone formation. We further fabricated a Gel-vHA@oe-DFSC scaffold to deliver engineered DFSCs in a rat periodontitis model, which protected cell viability under oxidative stress and facilitated periodontal regeneration. In summary, our findings identify IGFBP5 as a critical regulator of stem cell senescence, highlighting its value as both a molecular indicator for detecting cellular senescence and a therapeutic target for age-related periodontal bone loss (Fig. 9).

The decline in stemness of aging MSCs contributes to multi-organ dysfunction and disease progression, while the development of minimally invasive stem cell therapies for aging-related diseases is a key focus of current research.^5^ Among DSCs, DFSCs are unique as they are obtained at the earliest developmental stage of the tooth prior to eruption, and exhibit lower differentiation status, making DFSCs a highly promising candidate for regenerative medicine.^11^ Beyond their role as a therapeutic seed cell, DFSCs, as a core component of the adult periodontal stem cell pool, persist at the cementum-alveolar bone interface of the periodontal ligament (PDL) in the coronal and middle regions of the root.^32^ They are responsible for maintaining periodontal homeostasis and mediating injury repair, and the decline in their function induced by senescence directly impairs periodontal tissue renewal and bone regeneration.^33^ Consequently, DFSCs are being actively investigated for their potential in cell and gene therapies for a range of human diseases, especially periodontitis.^34^ However, significant challenges remain regarding their in vivo engraftment efficiency, controlled differentiation, and potential tumorigenicity, all of which necessitate additional rigorous investigation.^35^

Cellular senescence is broadly categorized into two types: RS caused by intrinsic physiological factors, and premature senescence, induced by external stressors such as H_2_O₂.^36^ That’s why we constructed both OSS and RS models of DFSCs initially. While both models exhibited senescence and oxidative stress, as detected by SA-β-Gal staining and 2’,7’-Dichlorodihydrofluorescein diacetate (DCFH-DA) assay, respectively, they showed distinct patterns in the intensity and dynamics of these markers. However, GO analysis revealed that 12 of the top 20 enriched terms were shared and IGFBP5 was ranked among the top 10 most significant DEGs in both aging models compared to YC. Given this substantial overlap in senescence-associated molecular signatures, the OSS model serves as a robust and representative system for investigating the mechanisms of DFSC senescence.

Our present study found that DFSCs possessed strong osteogenic differentiation potential, while their cellular functions were compromised under oxidative stress conditions, which accelerated senescence and likely contributed to tissue engineering failure. Previously, researchers have explored the mechanism and function of stem cell senescence and revealed its promising application in improving health.^3^ A major challenge in advancing stem cell therapy remains how to direct the differentiation of DFSCs into specific bone structures by regulating relevant cellular signaling pathways.^37^ Here, our research identified IGFBP5 as a core regulator of DFSC senescence, as it consistently showed significant expression differences in senescent cells regardless of the specific stressor that induced the aging process. Further experiments with IGFBP5-depleted and IGFBP5-overexpressing DFSCs confirmed that IGFBP5 could reverse H₂O₂-induced senescence in human primary DFSCs, which suggests that targeting IGFBP5 may offer a potential strategy for anti-aging treatments aimed at reducing age-related bone loss.

Among the IGFBP family, IGFBP5 is the most evolutionarily conserved, with a dynamic range of IGF-dependent and -independent functions.^17^ Notably, previous studies have demonstrated that local application of IGFBP5 protein enhanced periodontal tissue regeneration via increasing the migration and cell proliferation of MSCs.^38,39^ Nevertheless, it remains unclear whether IGFBP5 overexpression promotes osteogenesis in senescent DFSCs, and the underlying mechanism requires further elucidation. Our RNA-seq analysis revealed that under OSS, DFSCs overexpressing IGFBP5 exhibited upregulation of ALP and multiple collagen types, suggesting enhanced osteogenic potential. Notably, OCN levels in the OSS+oe-IGFBP5 group were comparable to those in the YC group, whereas RUNX2 expression remained relatively lower. While RUNX2 is typically regarded as an early transcription factor that initiates osteogenesis, OCN serves as a late-stage marker indicative of terminal maturation and mineralization.^40^ This differential expression pattern suggests the involvement of independent pathways that regulate the osteogenesis of DFSCs,^41^ potentially reflecting either a preferential rescue of late-stage mineralization by IGFBP5 or a partial reactivation of the osteogenic program in senescent cells without full restoration of early RUNX2-mediated signals. Moreover, collagen expression has been confirmed to strongly correlate with periodontal regeneration.^42^ In the oe-IGFBP5 group, type I collagen, localized in the extracellular region of the root furcation, was expressed at levels over two times higher, while type XII collagen, which is mainly present in the PDL, showed a more than 3.8-fold increase.^43^ The regenerative advantage of IGFBP5-overexpressing DFSCs was further corroborated in a rat model, where Masson staining revealed abundant blue-stained collagen fibers, indicating the critical role of collagen in promoting periodontal regeneration.

While IGFBP5 has been previously shown to modulate cell differentiation as a secreted factor through the Akt and JNK/MAPK pathway,^44,45^ its interaction with Wnt signaling remains less understood. To date, findings regarding IGFBP5-Wnt regulation have been confined to the Wnt/β-catenin pathway, and their relationship in MSCs remains unexplored.^46,47^ Our KEGG enrichment analysis showed that the Wnt signaling pathway was among the top three enriched terms. However, most regulators associated with the canonical Wnt/β-catenin pathway showed no significant changes in gene expression. Among the Wnt ligands, WNT2, WNT5B, WNT10B, and WNT16 were downregulated upon IGFBP5 overexpression, with only WNT2 as a canonical ligand.^48^ In contrast, key factors of the two non-canonical Wnt pathways were significantly altered, prompting further validation by PCR and Western blot. Our results demonstrated that IGFBP5 overexpression modulated the non-canonical Wnt pathways, accompanied by the downregulation of WNT5B and c-Jun. This is significant because c-Jun is a major substrate of JNK, a key kinase in the non-canonical Wnt signaling cascade.^49^ Accumulating evidence has established that JNK/c-Jun signaling is a critical positive regulator of cellular senescence.^50^ Activation of this pathway has been shown to promote senescence through multiple mechanisms, including the upregulation of P53 and P21, induction of the senescence-associated secretory phenotype, and mediation of stress-induced premature senescence under oxidative stress.^51^ Notably, elevated expression and phosphorylation of c-Jun are frequently observed in senescent cells and correlate with the establishment and maintenance of the senescent growth arrest state.^52,53^ In our model, the observed reduction in c-Jun levels following IGFBP5 overexpression suggests a suppression of JNK/c-Jun-mediated pro-senescence signaling. This interpretation is consistent with the enhanced proliferative and osteogenic capacity of IGFBP5-overexpressing DFSCs, as sustained JNK activity has been directly linked to growth arrest and senescence in various stem cell populations.^54^

Furthermore, the addition of WNT5B protein reversed the osteogenic effects of oe-IGFBP5 DFSCs, suggesting a functional antagonism between IGFBP5 and WNT5B. This antagonistic relationship was further supported by transcriptomic data showing an inverse correlation between IGFBP5 and WNT5B in both OSS and RS models, and WNT5B neutralization could partially rescue the senescent phenotype in RS cells. While these data collectively suggest that the IGFBP5-WNT5B regulatory axis may be functionally conserved under aging conditions, they do not distinguish whether WNT5B upregulation is a driver or a consequence of the senescent state. The observation that WNT5B neutralization partially alleviates senescence is consistent with both possibilities. Future studies investigating the induction of WNT5B during senescence, as well as genetic manipulation of WNT5B in young cells, would help clarify its precise hierarchical position in this pathway. Additionally, the upstream regulatory mechanisms underlying IGFBP5 downregulation in senescent DFSCs, such as promoter hypermethylation, repression by p53/NF-κB, or senescence-associated miRNAs, remain to be explored and represent important directions for future research.^55,56^

The periodontium is a functional unit composed of four interconnected tissues, gingiva, cementum, alveolar bone, and the PDL, that together anchor the tooth and maintain oral homeostasis.^57^ Given this complexity, biomaterial scaffolds are frequently combined with stem cells or bioactive molecules to promote new bone formation at defect sites. Beyond scaffold-based approaches, other therapeutic platforms have recently been explored for periodontal treatment. For instance, advanced computer-aided drug design strategies for multifunctional nanoinhibitors and environment-adaptive microneedle systems have emerged as promising precision tools for managing oral diseases.^58,59^ GelMA, a photopolymerizable hydrogel derived from collagen, offers significant advantages for bone tissue engineering due to its high biocompatibility, favorable cell adhesion properties, and tunable biodegradability.^24^ Hydroxyapatite (HA), the primary inorganic component of native bone, also exhibits excellent biocompatibility. To improve its integration into composite scaffolds, silane coupling agents are widely used for surface modification of HA nanoparticles, which enhances particle dispersion within a polymer matrix.^26^ Based on these principles, we developed Gel-vHA@oe-DFSC, a composite scaffold designed to deliver stem cells for enhanced periodontal regeneration. Interestingly, previous studies have identified that IGFBP5 contains a primary heparin-binding domain that specifically interacts with HA.^60^ We propose that this specific binding enhances the scaffold’s mechanical integrity, retards its degradation, and establishes a sustained, localized microenvironment to direct stem cell fate for improved periodontal regeneration. More preclinical and clinical trials are required for further investigation and applications.

In conclusion, our study first established the pivotal role of IGFBP5 in alleviating DFSCs' senescence, a process involving modulation of the non-canonical Wnt axis. IGFBP5 expression could serve as a molecular indicator of DFSC senescence in ex vivo expanded cells prior to transplantation, while the Gel-vHA@oe-DFSC scaffold provides a clinically adaptable platform for localized stem cell delivery. The clinical significance of this study lies in demonstrating that rejuvenating senescent DFSCs prior to implantation, rather than merely transplanting naive stem cells, markedly enhances periodontal bone regeneration. Rejuvenating aged stem cells through targeted interventions like IGFBP5 represents a promising therapeutic strategy for systemic aging-related bone disorders by simultaneously alleviating cellular senescence and restoring osteogenic potential. Future work should focus on validating the IGFBP5-WNT5B regulatory axis in clinical DFSC isolates from aged patients, optimizing the hydrogel formulation for minimally invasive intraoral injection, and assessing long-term safety and efficacy in large-animal periodontal defect models.

## Materials and methods

### Cell culture

Human dental follicle stem cells (hDFSCs) were obtained from freshly extracted third molars of healthy, young individuals under 18 years of age. Rat dental follicle stem cells (rDFSCs) were harvested from neonatal SD rats aged 3–5 days and cultured. DFSCs at P3 were used in cellular experiments, with DFSCs at P12 serving as the RS group. Cells in the OSS group were incubated with 150 μmol/L H₂O₂ for 24 h. Osteogenic differentiation was induced using 10 nM dexamethasone (Sigma), 50 µg/mL ascorbic acid (Sigma), and 5 mmol/L β-glycerophosphate (Sigma).

### SA-β-gal staining

Cells seeded in culture plates were fixed with the paraformaldehyde (PFA) for 10-15 min at room temperature. After fixation, cells were washed 2-3 times with PBS. Subsequently, the cells were incubated with a freshly prepared SA-β-gal staining solution at 37 °C in a low CO₂ environment and protected from light. Stained cells were observed under a light microscope for the presence of blue coloration.

### RT-qPCR

Total RNA was extracted using Trizol reagent. cDNA was synthesized from 1 μg RNA with HiScript III RT SuperMix for qPCR (Vazyme, China). qRT-PCR was performed using Taq Pro Universal SYBR qPCR Master Mix (Vazyme, China) with β-Actin as the internal control. Gene expression was calculated by the 2^−^^ΔΔCT^ method. All experiments were conducted in triplicate.

### Cellular ROS detection

Intracellular ROS were detected using the fluorescent probe DCFH-DA (Beyotime, China). After treatments, cells were incubated with 20 μmol/L DCFH-DA at 37 °C for 30 min and washed with PBS. ROS production was immediately examined and visualized under a fluorescence microscope.

### Cell viability assay

For the CCK-8 assay, cells were seeded in 96-well plates. After 24 h of culture, the medium was replaced with various formulations for another 24 h. Subsequently, the cells were incubated with a fresh medium containing 10% (v/v) CCK-8 reagent (Beyotime, China) in the dark for 2 h, and the absorbance was measured. The cell viability was calculated. To get a cell proliferation curve, cells were continuously cultured for 6 days, and the OD value was measured daily using the CCK-8 assay following the same incubation procedure.

### Transwell migration assay

Cell migration was assessed using Transwell chambers (Labselect, China). Briefly, DFSCs suspended in serum-free medium were seeded into the upper chambers. Complete medium was added to the lower chamber as a chemoattractant. After 48 h of incubation, non-migrated cells on the top surface were removed. The migrated cells on the bottom surface were fixed with 4% PFA, stained with 0.2% crystal violet, and imaged under a microscope.

### Alizarin Red S staining

After 14 days of osteogenic induction, DFSCs were fixed and stained with 2% alizarin red solution (pH 4.2) for 10 min to detect calcium deposits. After washing, the mineralized nodules were observed and imaged under an inverted microscope. After imaging, the mineralized nodules were dissolved with 10% cetylpyridinium chloride in PBS for 30 min at room temperature. The absorbance of the solution was measured at 550 nm.

### ALP staining

After 14 days of osteogenic induction, DFSCs were fixed and stained using a BCIP/NBT ALP Color Development Kit (Beyotime, China) according to the manufacturer’s instructions. Stained cells were visualized and imaged under an inverted microscope. ALP activity was quantified using an ALP Assay Kit (Nanjing Jiancheng, China) and the absorbance at 520 nm was measured. ALP activity per gram of protein was calculated.

### RNA-Seq

Two independent RNA-seq experiments were conducted with three biological replicates per group: the first compared OSS and RS groups against the YC control; the second compared OSS+oe-IGFBP5 against OSS+Vehicle. Total RNA was extracted using Trizol Reagent. RNA concentration was measured with a Nanodrop ND-2000 (Thermo Fisher, USA), and integrity was assessed using an Agilent 4150 system (Agilent, USA). Strand-specific cDNA libraries were prepared from poly(A)-enriched mRNA using the NEBNext® Ultra™ RNA Library Prep Kit for Illumina® and sequenced on a Novaseq 6000 platform (Illumina, USA) by Shanghai Applied Protein Technology Co., Ltd (Shanghai, China). Raw data were processed through the following pipeline: Quality control was performed using fastp (v0.23.2). Clean reads were aligned to the reference genome using HISAT2 (v2.2.1). Gene expression levels were quantified with FeatureCounts (v2.0.3). Differential expression analysis was performed using DESeq2 (|log₂ FC| > 1, *P*adj<0.05). Functional enrichment analysis (GO and KEGG) was conducted using clusterProfiler.

### Lentiviral infection

Knockdown and overexpression lentiviruses targeting IGFBP5, along with a blank vehicle control, were purchased from Hanbio, China. DFSCs were infected with lentiviruses at an MOI of 25. After 24 h, the virus-containing medium was replaced with fresh medium. Infection efficiency was validated by qRT-PCR at 48 h or Western blot at 72 h post infection.

### Western blot

Proteins were extracted using protein extraction reagent (Vazyme, China) and quantified with a BCA assay (Vazyme, China). Protein samples of 20 μg were separated by SDS-PAGE and transferred onto PVDF membranes. After blocking with 5% non-fat milk for 1 h at room temperature, membranes were incubated overnight at 4 °C with the following primary antibodies: anti-IGFBP5 (1:1 000, rabbit, CST, 10941), anti-β-Catenin (1:1 000, rabbit, CST, 8480), anti-phospho-β-Catenin (1:1 000, rabbit, CST, 9561), anti-active-β-Catenin (1:1 000, rabbit, CST, 19807), anti-phospho-GSK3β (1:1 000, rabbit, CST, 5558), anti-WNT5A/B (1:1 000, rabbit, CST, 2530), anti-c-JUN (1:1 000, rabbit, CST, 9165), anti-phospho-c-JUN (1:1 000, rabbit, CST, 3270), anti-ALP (1:5 000, rabbit, HUABIO, ET1601-21), anti-RUNX2 (1:500, rabbit, HUABIO, ET1612-47), anti-OCN (1:1 000, rabbit, abcam, ab93876), anti-Col I (1:1 000, rabbit, HUABIO, ET1609-68), anti-Periostin (1:1 000, rabbit, abcam, ab219056), anti-P21 (1:500, rabbit, ZenBio, R381102), anti-P16 (1:500, rabbit, HUABIO, ET1608-62), and anti-Tubulin (1:1 000, mouse, abcam, ab7291). Membranes were then incubated with HRP-conjugated secondary antibodies at room temperature for 1 h. Protein bands were visualized using SuperSignal™ West Femto Reagent (Thermo Scientific, USA). Protein expression levels were normalized to Tubulin using ImageJ software.

### Live/Dead staining

Cells were treated according to the experimental design. After treatments, cells were co-stained with Calcein AM and PI in the dark at 37 °C for 30 min. Following incubation, cells were washed with PBS to remove excess dye. Fluorescent images were immediately captured using a fluorescence microscope. Viable cells (green fluorescence) and dead cells (red fluorescence) were quantified using ImageJ software.

### Cell cycle detection

Cells were harvested and fixed in 70% cold ethanol overnight at 4 °C. Fixed cells were then incubated with PI/RNase Staining Buffer (Beyotime, China) at 37 °C for 30 min in the dark. Cell cycle distribution was analyzed using a BD flow cytometer, and data were processed with FlowJo software.

### Preparation and characterization of vHA

vHA was synthesized as follows: 5 mL of TEOS and a corresponding amount of silane coupling agent YDH-151 were separately added dropwise into 100 mL of acidified aqueous ethanol solution (pH 4.0). After hydrolyzing for 2 h under stirring, a slurry containing 5 g of pristine nHA (Macklin, 200 nm H875580, China) was introduced, and the reaction continued for 12 h. The mixture was then adjusted to pH 9.0 using 10% NaOH. The resulting product was thoroughly washed with ethanol and water, and dried. The synthesized vHA, alongside unmodified nHA, was characterized using multiple techniques: chemical structure via FT-IR (Thermo Fisher, Nicolet iS10, USA), crystal phase via XRD (Rigaku, Ultima IV, Japan), surface elemental composition via XPS (Thermo Fisher, K-ALPHA, USA), morphology via SEM (JSM-IT700HR, JEOL, Japan) and TEM (Hitachi, HT7800, Japan), and particle size distribution via DLS (Malvern, Zetasizer Nano ZS90, UK).

### Preparation of Gel-vHA@oe-DFSC

A 0.25% (w/v) LAP solution in PBS was prepared as the photoinitiator solution. GelMA powder (Engineering For Life, China) was dissolved in this solution to form a 10% (w/v) precursor. nHA or vHA nanoparticles were added to achieve final concentrations of 0.1% or 1% (w/v), forming Gel-nHA or Gel-vHA composites. After thorough mixing, an oe-DFSC cell suspension was incorporated. The mixture was photo-crosslinked under 405 nm light for 20 s, and the resulting cell-laden hydrogel was designated as the Gel-nHA@oe-DFSC and Gel-vHA@oe-DFSC hydrogel.

### Characterization of hydrogel

For mechanical testing, the mixture was poured into customized molds and crosslinked under blue light to form standardized specimens. Rheological analysis was performed using a rheometer (Anton Paar, MCR302, China) with a fixed strain of 5% and a frequency sweep from 0.1 to 10 Hz. The compressive strength was measured on cylindrical specimens (*d* = 10 mm, *h* = 10 mm) using a universal testing machine (Shimadzu, AGS-X, Japan) at a crosshead speed of 2 mm/min. The microstructures of each hydrogel were observed using SEM at 20 kV with a gold coating obtained by a sputter coater. The swelling capacity and degradation profiles of Gel, Gel-nHA, and Gel-vHA hydrogels (*n* = 3) were evaluated in PBS (pH 7.4) at 37 °C. For the swelling test, the weight change of lyophilized samples was monitored after immersion in PBS over predetermined time intervals. For the degradation study, the weight loss of the lyophilized hydrogels was measured following incubation in PBS for 1, 2, 4, 7, 10, 20 and 30 days.

### Cytoskeleton staining and confocal microscopy

Cells were seeded on confocal dishes and subjected to the experimental treatments. After treatment, cells were fixed with 4% paraformaldehyde for 15 min and permeabilized with 0.25% Triton X-100. Following blocking with 5% BSA, F-actin was stained with FITC-phalloidin at 37 °C for 20 min, and nuclei were counterstained with DAPI. Fluorescence images were captured using a confocal microscope (Olympus, FV3000, Japan).

### Animal experiments

All animal experiments were approved by the Institutional Laboratory Animal Care and Use Committee of Sichuan University. Six-week-old male Sprague-Dawley (SD) rats (weight, 240 g ± 10 g; *n* = 6 per group) were housed under a 12 h light/dark cycle at 22 °C ± 2 °C and 55% ± 5% humidity. All animals were anesthetized with isoflurane gas and treated according to a modified ligature-induced periodontitis model. Briefly, rats were subjected to ligation of the second molar for 1 month and then received stem cell therapy or a sham operation. All rats were sacrificed on day 30 for sample collection and subsequent analysis.

### Micro-CT analysis

Alveolar bone samples were fixed and scanned using a high-resolution micro-CT system (µCT 45, Scanco, Switzerland). The scanning parameters were set with a voxel size of 20 μm. Three-dimensional reconstructions were generated, and bone structural parameters including BMD, BV/TV and linear bone loss measurements were quantified using CTAn software.

### Histological observation

After fixation and decalcification, bone samples were embedded in paraffin and sectioned at 5 μm thickness. Sections were stained with H&E and Masson’s Trichrome using standard protocols. For IHC, sections underwent antigen retrieval and blocking before incubation with primary antibodies at 4 °C overnight: anti-RUNX2 (1:500, rabbit, HUABIO, ET1612-47), anti-Col I (1:1 000, rabbit, CST, 72026), anti-OCN (1:200, rabbit, abcam, ab93876), anti-IGFBP5 (1:200, rabbit, HUABIO, ER1911-14), and anti-WNT5A/B (1:200, rabbit, Proteintech, 55184-1-AP). Detection was performed using HRP-conjugated goat anti-rabbit IgG and DAB development, followed by hematoxylin counterstaining. All sections were examined under a light microscope. For GLB1/beta-galactosidase immunofluorescence, sections were incubated with anti-GLB1 (1:1 000, rabbit, HUABIO, HA721562) overnight at 4 °C, followed by incubation with a fluorophore-conjugated secondary antibody and DAPI counterstaining. Sections were mounted and examined under a fluorescence microscope.

### Statistical analysis

Statistical analyses were processed by GraphPad Prism 8. One-way analysis of variance (ANOVA) and Student’s *t*-test were applied to evaluate the significance. All data were expressed as mean ± SD; *P* < 0.05 was considered statistically significant, *P < 0.05, **P < 0.01, ***P < 0.001.

## Supplementary information

Supplementary Materials

## Data Availability

The data have been released and are publicly available in the NCBI Sequence Read Archive (SRA) under BioProject accession number PRJNA1480452 (BioSample accession numbers: SAMN61623109–SAMN61623123, SAMN61623578–SAMN61623579). All other data supporting the findings of this study are available from the corresponding author upon reasonable request.
