# Supplementary material for: IGFBP5 alleviates periodontitis by reversing human dental follicle stem cell senescence via the non-canonical Wnt pathway
Source: Int J Oral Sci. 2026 Sep 15;18:63. doi: 10.1038/s41368-026-00463-2 (PMC13575160; doi:10.1038/s41368-026-00463-2)
Supplement: Supplementary file 1 [file 41368_2026_463_MOESM1_ESM.pdf]

Supplementary Materials

IGFBP5 alleviates periodontitis by reversing human dental follicle stem cell senescence via the non-canonical Wnt pathway

Yuru Wang[1,2], Yue Wang[1,2], Yuguo Dai[3], Weihua Guo[4], Jun Liu[1,2,†]

1 State Key Laboratory of Oral Diseases & National Center for Stomatology & National Clinical Research Center for Oral Diseases, West China Hospital of Stomatology, Sichuan University, Chengdu 610041, Sichuan, People's Republic of China.

2 Department of Orthodontics, West China Hospital of Stomatology, Sichuan University, Chengdu 610041, Sichuan, People's Republic of China.

3 Division of Restorative Dental Sciences, Faculty of Dentistry, The University of Hong Kong, Hong Kong SAR 999077, People's Republic of China.

4 Yunnan Key Laboratory of Stomatology & Department of Pediatric Dentistry, The Affiliated Stomatology Hospital, Kunming Medical University, Kunming 650500, Yunnan, People's Republic of China.

Supplementary Figure 1

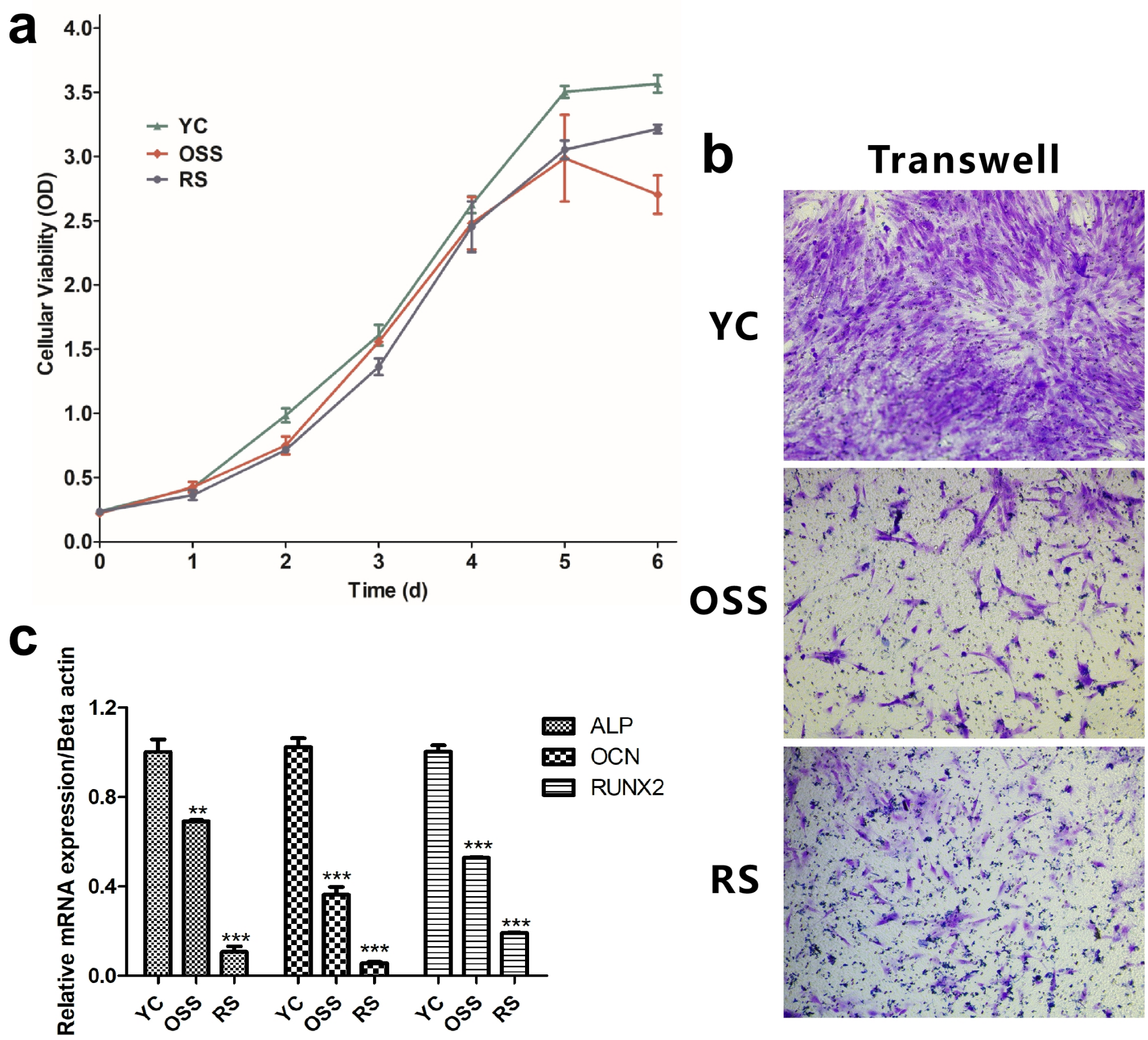

Supplementary Figure 1. Impaired stem cell function in senescent dental follicle stem cells (DFSCs). **a** Cell proliferation was assessed by CCK-8 assay over a 6-day period. **b** Representative images of the Transwell migration assay in each group. **c** Quantitative real-time PCR (qRT-PCR) analysis of the mRNA expression levels of ALP, OCN, and RUNX2. Data are presented as the mean ± SD (n=3). $**P < 0.01$; $***P < 0.001$. Abbreviations: YC, young cells; OSS, oxidative stress-induced senescence; RS, replicative senescence.

Supplementary Figure 2

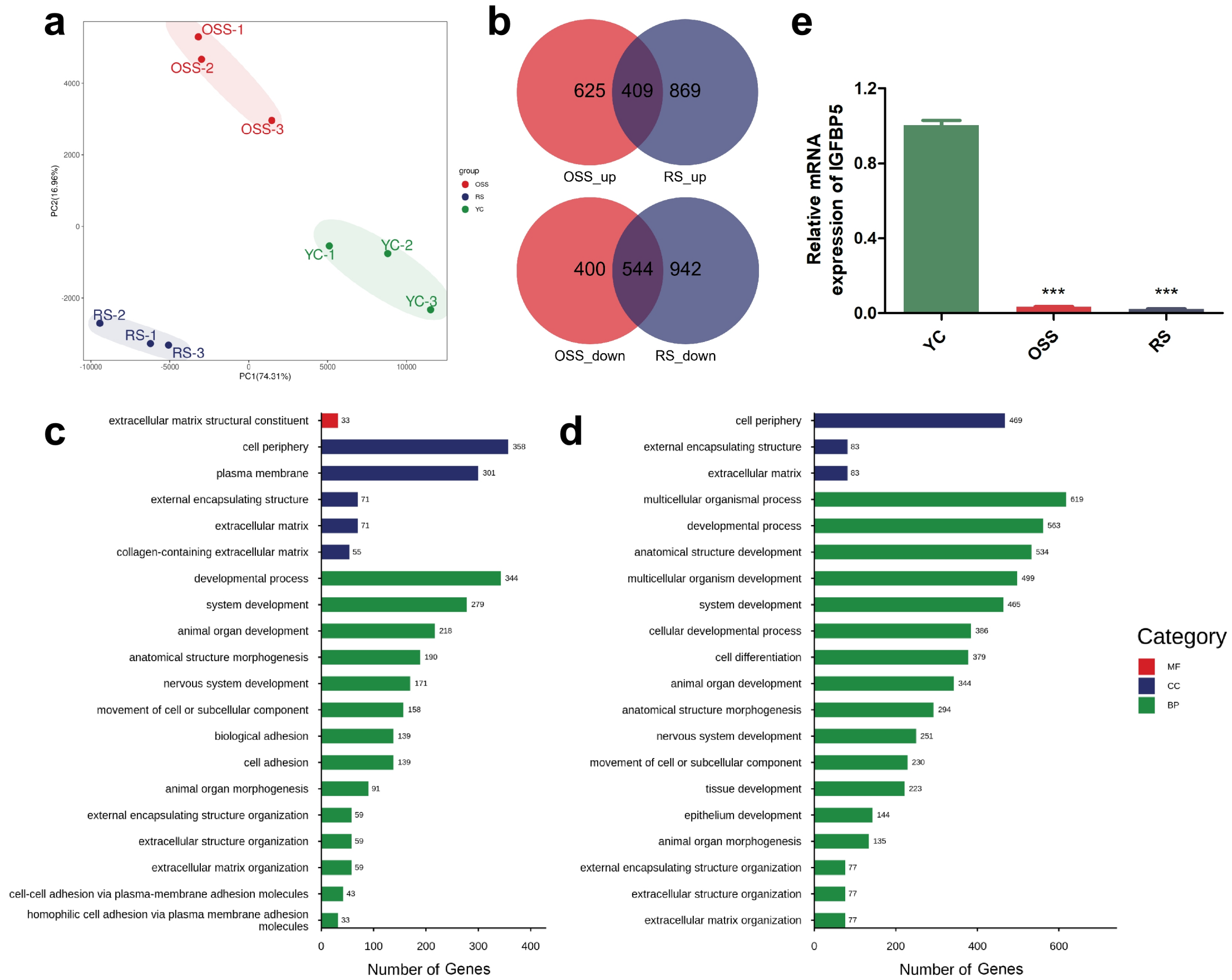

Supplementary Figure 2. Transcriptomic analysis of DFSCs under different senescence states. **a** Principal component analysis (PCA) of mRNA expression profiles in YC, OSS, and RS groups. **b** Venn diagram showing the number of commonly upregulated and downregulated genes across OSS and RS models compared to YC. **c** Gene Ontology (GO) enrichment analysis of the top 20 enriched terms based on differentially expressed genes (DEGs) between OSS and YC. **d** GO enrichment analysis of the top 20 enriched terms based on DEGs between RS and YC. **e** qRT-PCR analysis showing the expression of IGFBP5 under senescence states. Abbreviations: MF, molecular function; CC, cellular component; BP, biological process.

Supplementary Figure 3

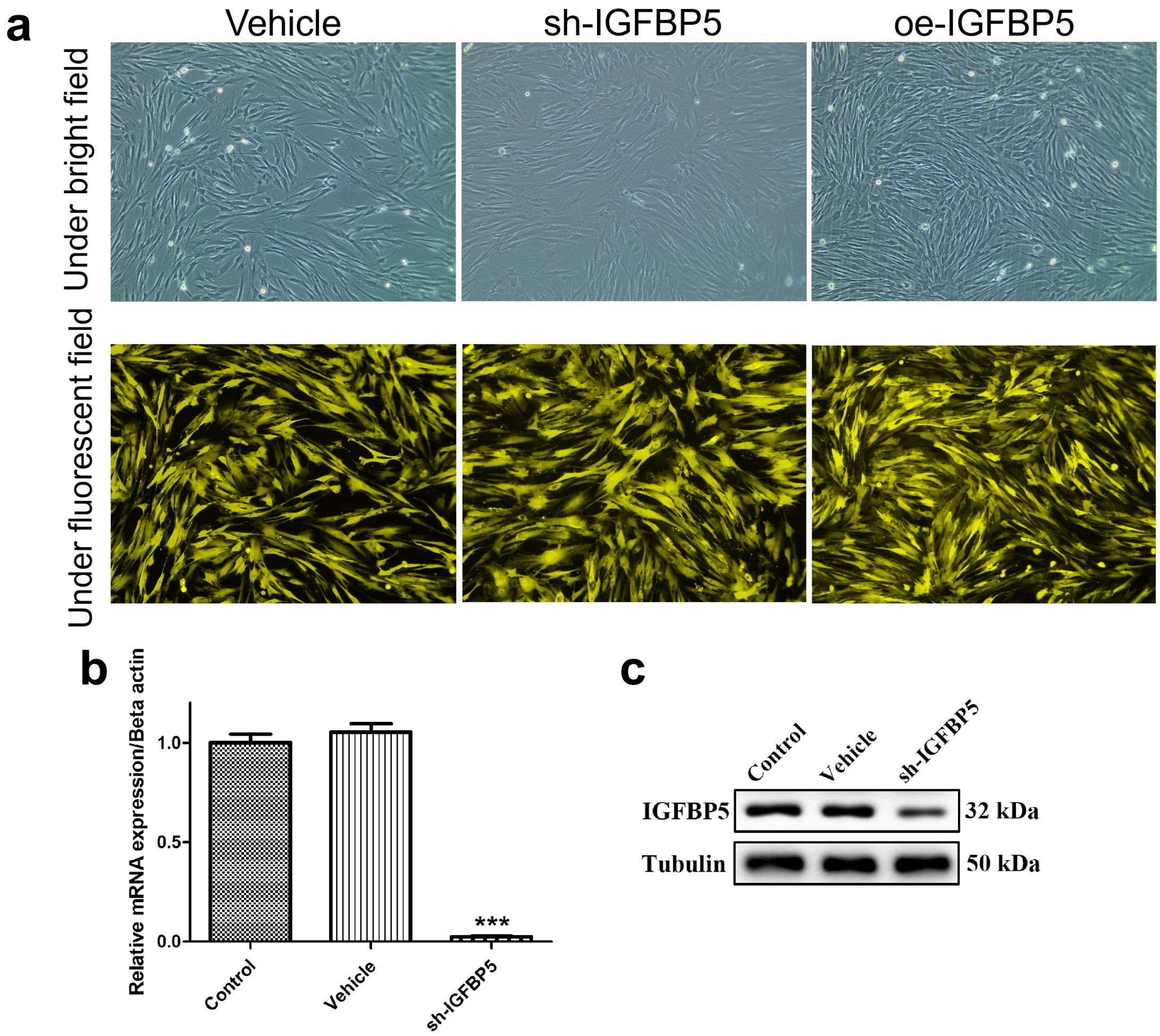

Supplementary Figure 3. Viral transduction of IGFBP5 knockdown and overexpression. **a** Representative bright-field and corresponding fluorescence images of Vehicle, sh-IGFBP5, and oe-IGFBP5 groups post viral transduction. **b** qRT-PCR analysis of IGFBP5 mRNA expression in Control, Vehicle, and sh-IGFBP5 groups. **c** Western blot analysis of IGFBP5 protein expression in Control, Vehicle, and sh-IGFBP5 groups. Data are presented as the mean ± SD (n=3). ***P < 0.001.

Supplementary Figure 4

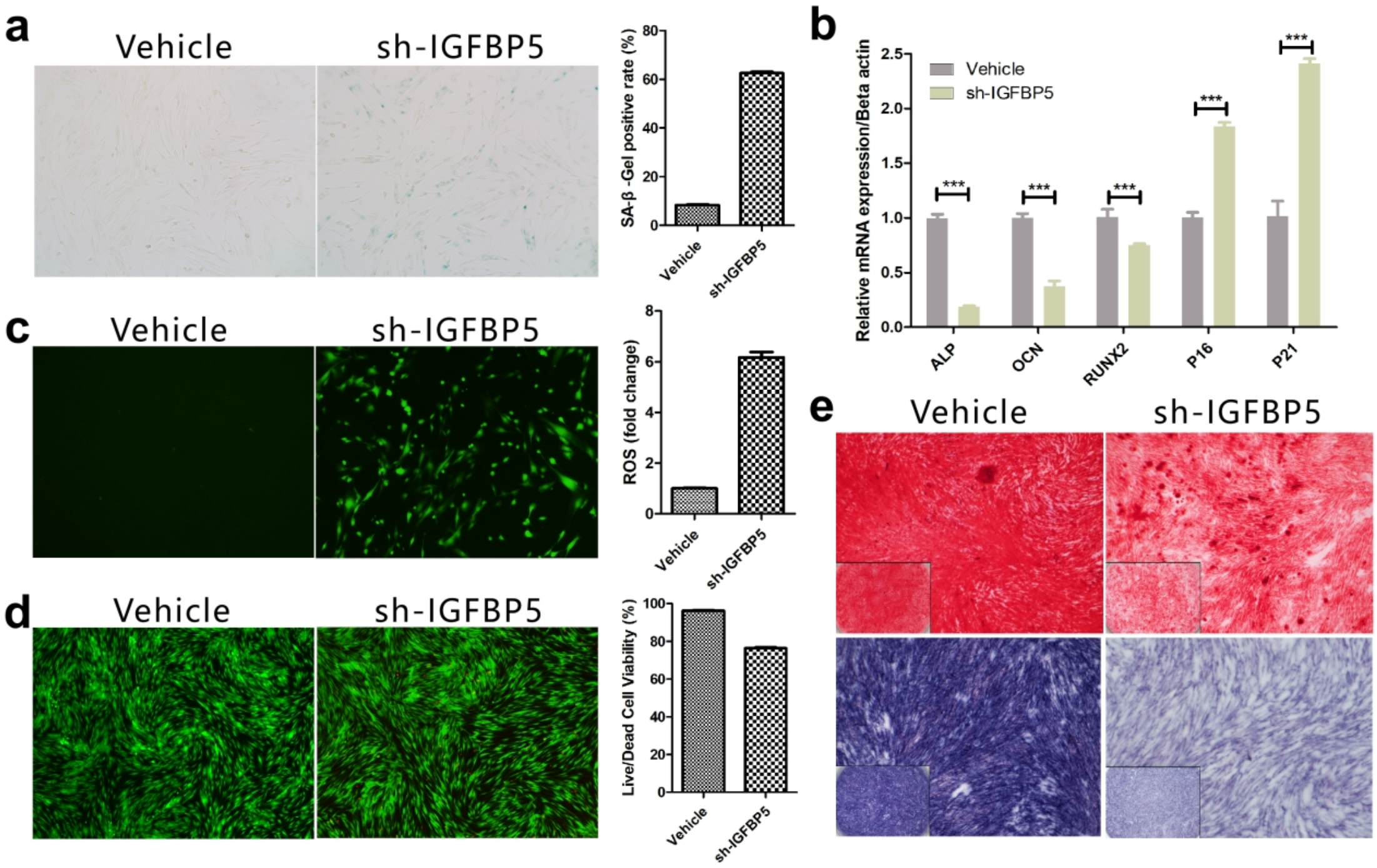

Supplementary Figure 4. IGFBP5 deficiency facilitated DFSCs senescence. **a** Senescence-associated β-galactosidase (SA-β-Gal) staining and quantification showing a higher percentage of senescent cells in the sh-IGFBP5 group. **b** qRT-PCR analysis of the mRNA expression levels of osteogenic-related genes (ALP, OCN and RUNX2) and aging-related genes (P16 and P21). **c** 2′,7′-Dichlorodihydrofluorescein diacetate (DCFH-DA) staining and quantification of fluorescence intensity showing increased intracellular reactive oxygen species (ROS) levels in the sh-IGFBP5 group. **d** Live/dead staining and quantification showing lower cell viability in the sh-IGFBP5 group. **e** Alizarin red and alkaline phosphatase (ALP) staining showing impaired osteogenic differentiation in the sh-IGFBP5 group. Data are presented as the mean ± SD (n=3). ***P < 0.001.

Supplementary Figure 5

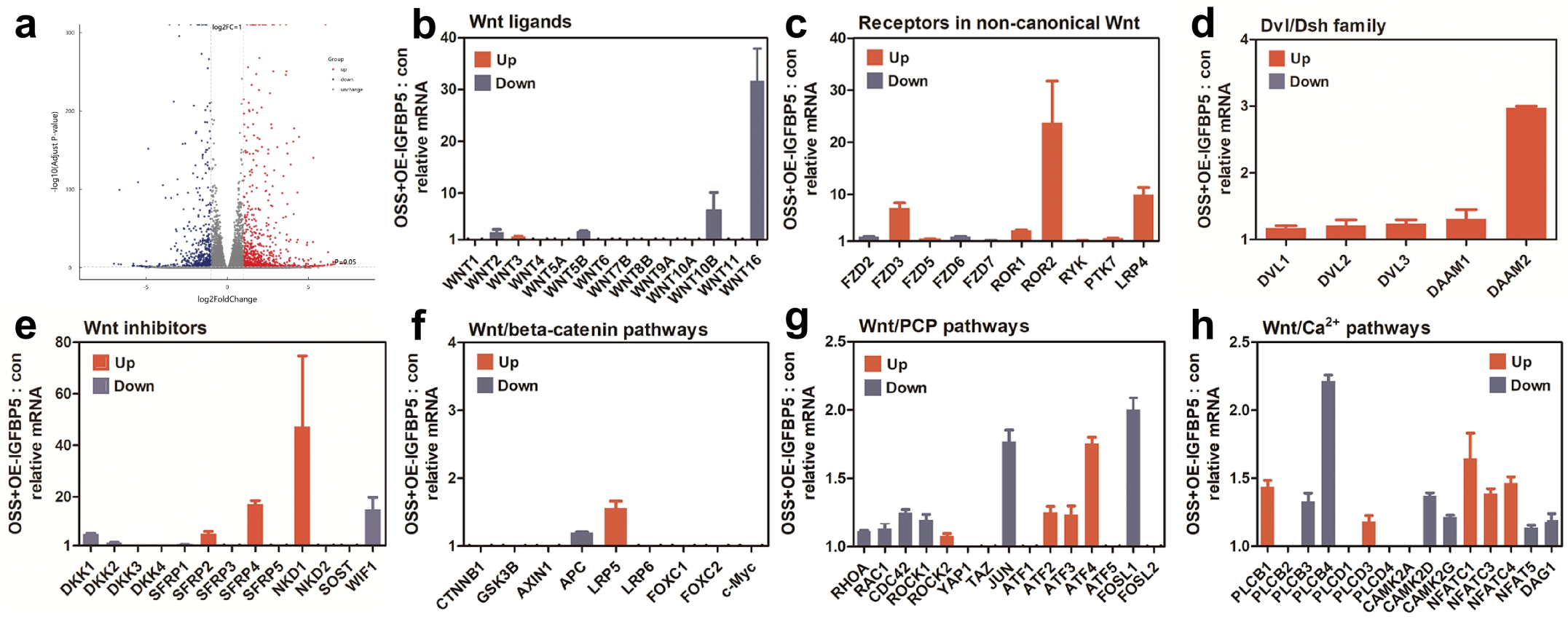

Supplementary Figure 5. DEGs between OSS+oe-IGFBP5 and OSS+Vehicle groups. **a** Volcano plot showing 796 upregulated (red) and 582 downregulated (blue) genes. **b** Fold change in DEGs related to Wnt ligands. **c** Fold change in DEGs related to receptors in the non-canonical Wnt pathway. **d** Fold change in DEGs related to the Dvl/Dsh family. **e** Fold change in DEGs related to Wnt inhibitors. **f** Fold change in DEGs related to the Wnt/β-catenin pathway. **g** Fold change in DEGs related to the Wnt/PCP pathway. **h** Fold change in DEGs related to the Wnt/Ca^{2+} pathway. Abbreviations: FZD, Frizzled; ROR, receptor tyrosine kinase-like orphan receptor; RYK, receptor-like tyrosine kinase; PTK7, protein tyrosine kinase 7; LRP, low-density lipoprotein receptor-related protein; DVL, Dishevelled; DAAM, Dishevelled-associated activator of morphogenesis; DKK, Dickkopf-related protein; SFRP, secreted frizzled-related protein; NKD, Naked cuticle homolog; SOST, Sclerostin; WIF1, Wnt inhibitory factor 1; CTNNB1, catenin beta-1; GSK3B, glycogen synthase kinase 3 beta; AXIN1, Axin-1; APC, adenomatous polyposis coli; LRP, low-density lipoprotein receptor-related protein; FOXC, forkhead box protein C; c-MYC, cellular Myc; RHOA, Ras homolog family member A; RAC1, Ras-related C3 botulinum toxin substrate 1; CDC42, cell division cycle 42; ROCK, Rho-associated protein kinase; YAP1, Yes1-associated transcriptional regulator; TAZ, tafazzin; JUN, Jun proto-oncogene; ATF, cyclic AMP-dependent transcription factor; FOSL, Fos-like antigen 1; PLCB, phospholipase C beta; PLCD, phospholipase C delta; CAMK, calcium/calmodulin-dependent protein kinase; NFATC, nuclear factor of activated T-cells, cytoplasmic; NFAT, nuclear factor of activated T-cells; DAG1, dystroglycan; PCP, planar cell polarity.

Supplementary Figure 6

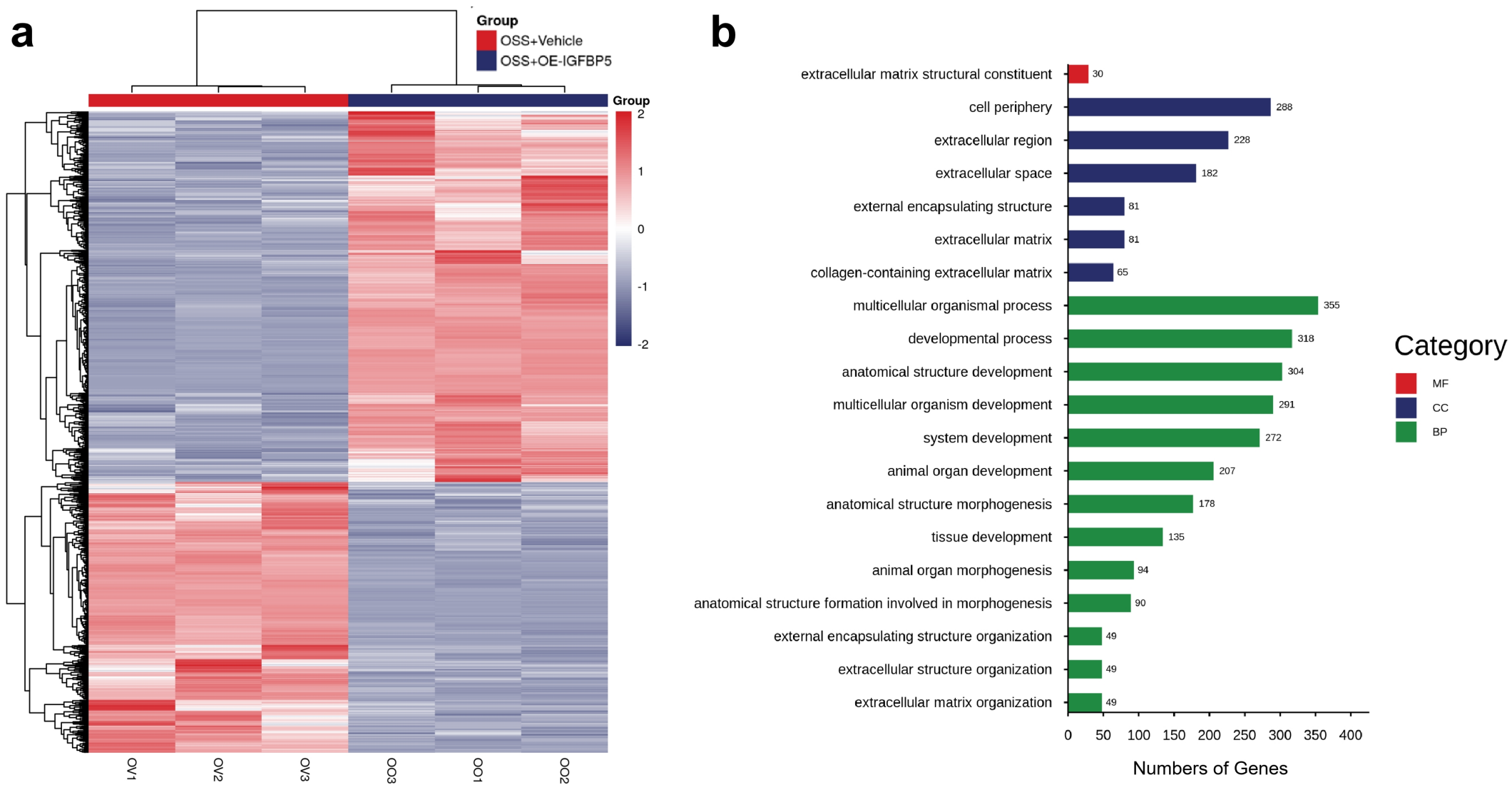

Supplementary Figure 6. RNA-seq analysis of DEGs and GO enrichment in IGFBP5-overexpressing versus vehicle-treated DFSCs. **a** Heatmap of RNA-seq results demonstrating the significantly differential expression in the IGFBP5-overexpressing condition. **b** GO analysis of the top 20 enriched terms based on DEGs.

Supplementary Figure 7

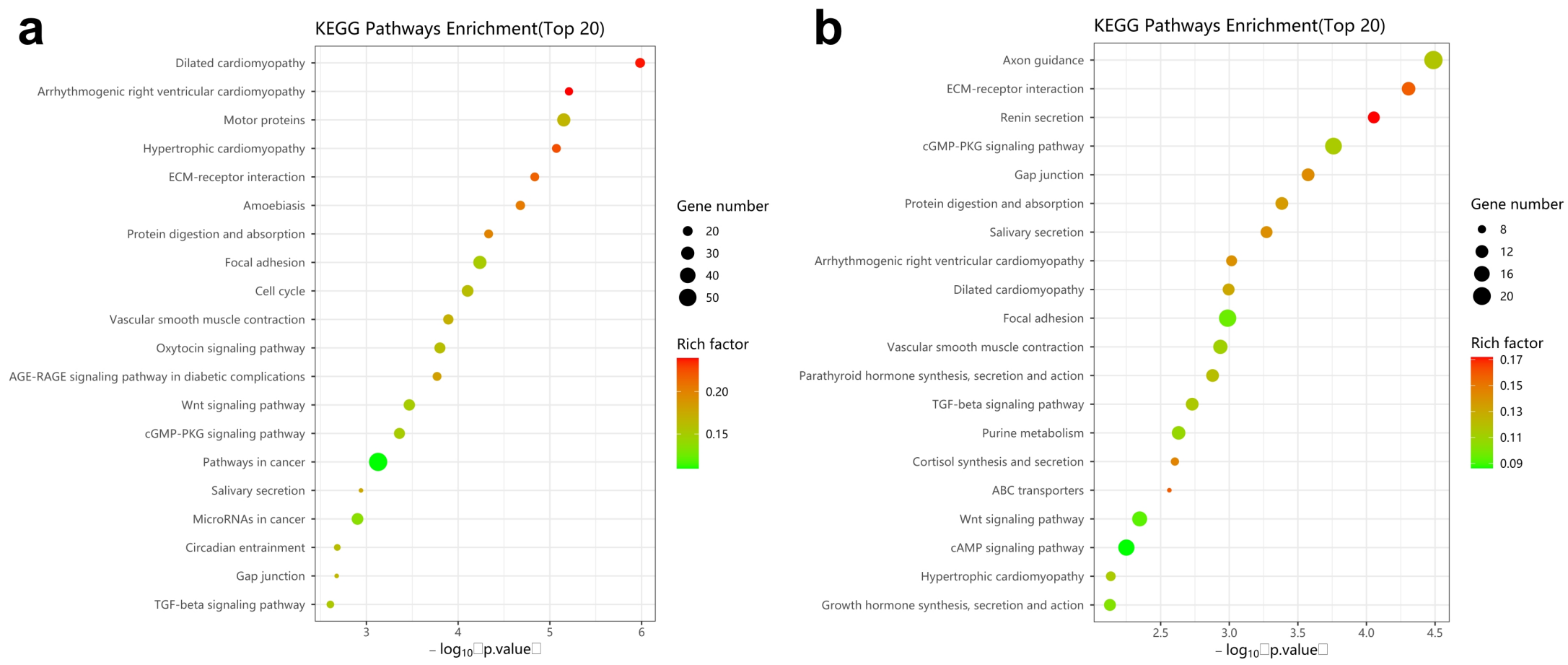

Supplementary Figure 7. Kyoto encyclopedia of genes and genomes (KEGG) enrichment analysis of the top 20 downregulated enriched terms based on DEGs in OSS and RS compared with YC. **a** Top 20 downregulated enriched terms in OSS compared with YC. **b** Top 20 downregulated enriched terms in RS compared with YC.

Supplementary Figure 8

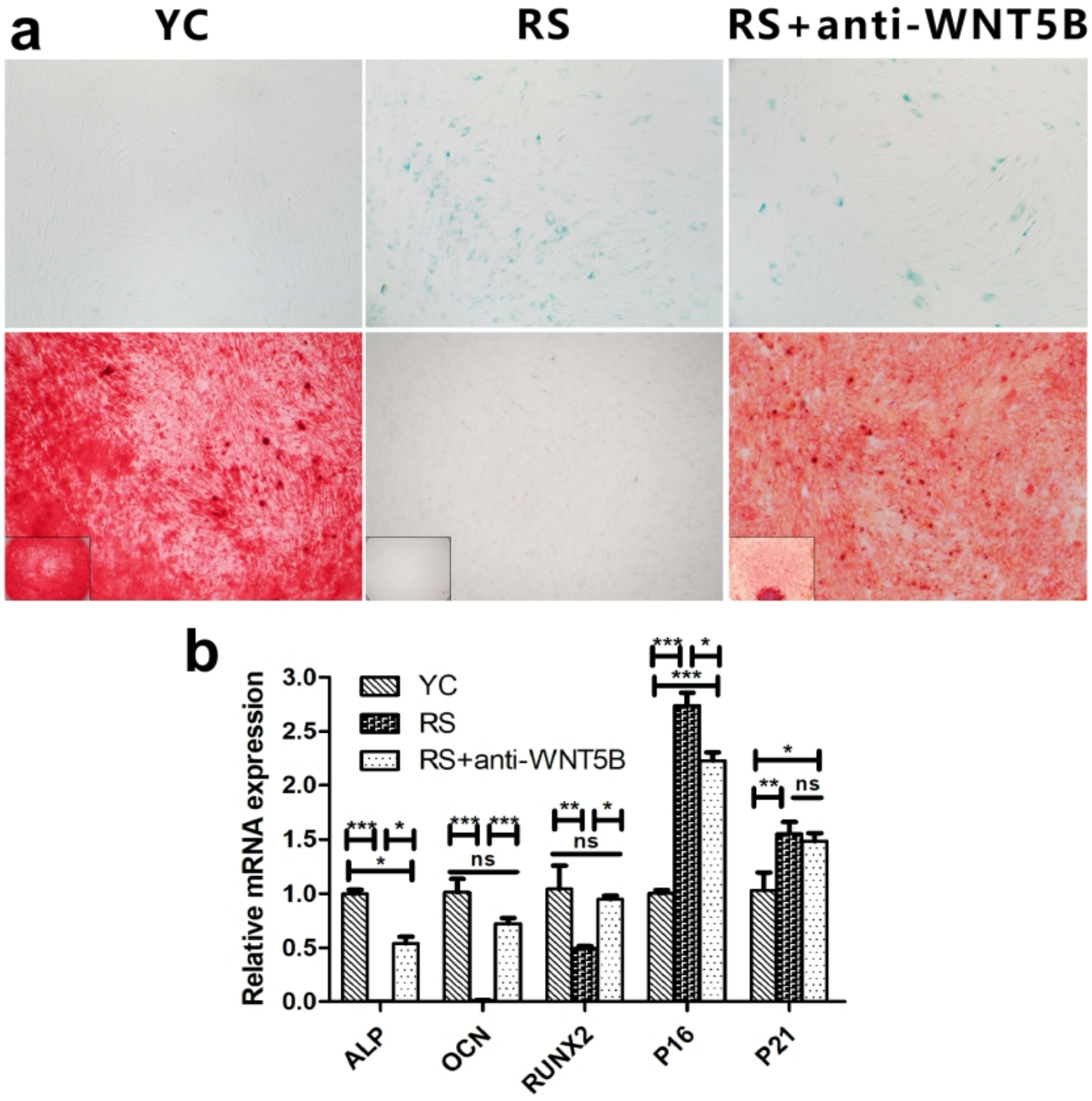

Supplementary Figure 8. Validation of WNT5B as a mediator of senescence and osteogenesis in the RS model. **a** SA-β-Gal staining and Alizarin red staining in RS cells treated with or without anti-WNT5B neutralizing antibody. **b** qRT-PCR analysis of the mRNA expression levels of senescence-associated genes (P16 and P21) and osteogenic-related genes (ALP, OCN, and RUNX2) in YC, RS, and RS+anti-WNT5B groups. Data are presented as the mean ± SD (n=3). ns, no significance; $*P < 0.05$; $**P < 0.01$; $***P < 0.001$.

Supplementary Figure 9

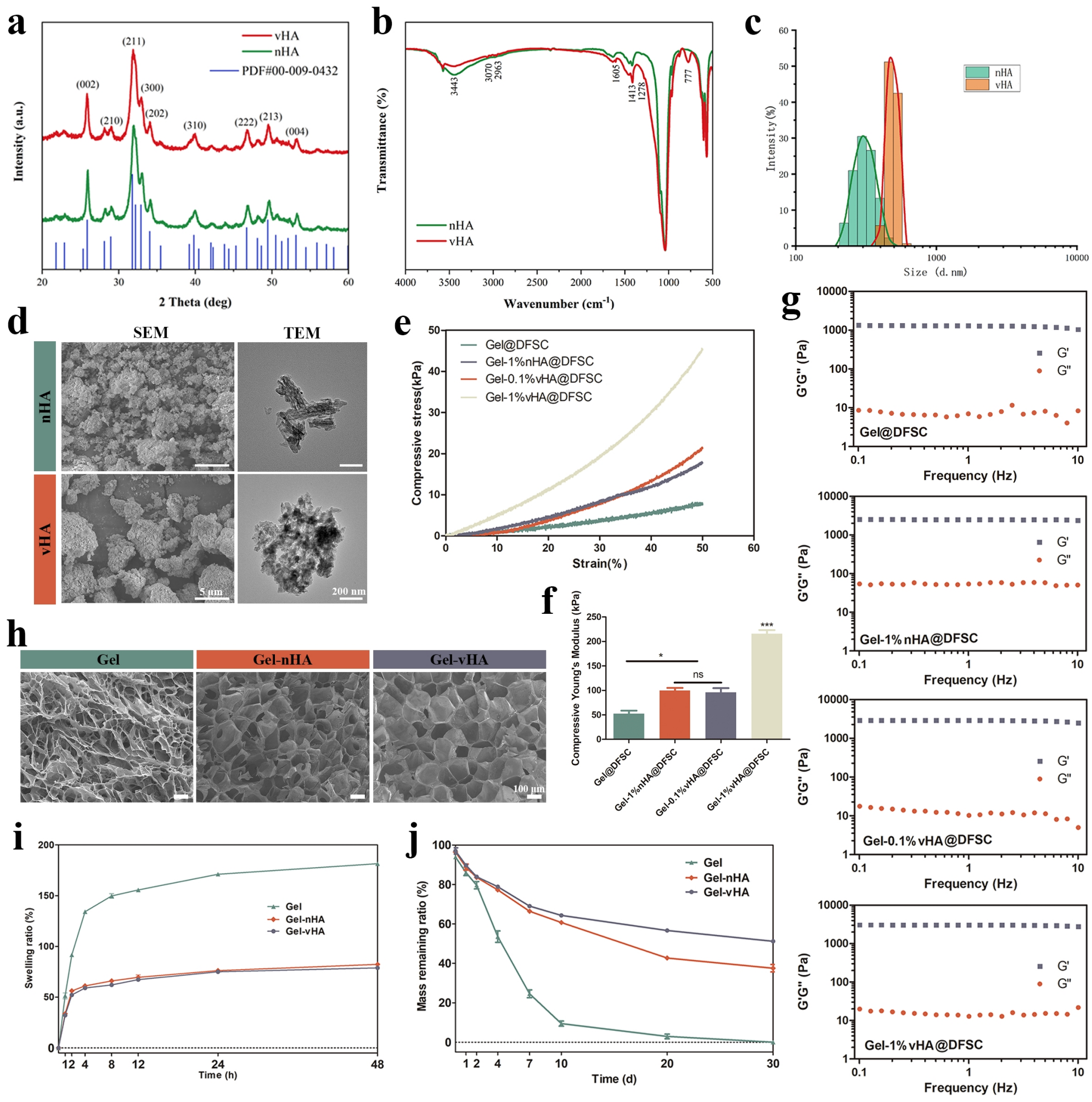

Supplementary Figure 9. Preparation and characterization of vinyl-modified nanohydroxyapatite (vHA) nanoparticles and Gel-vHA scaffolds. **a** X-ray diffraction (XRD) spectra of nanohydroxyapatite (nHA), vHA, and the standard spectrum of hydroxyapatite ($Ca_{10}(PO_4)_6(OH)_2$, PDF#00-009-0432). **b** Fourier transform infrared spectroscopy (FTIR) spectra of nHA and vHA to verify successful synthesis. **c** Size distribution of nHA and vHA measured by dynamic light scattering (DLS). **d** Representative scanning electron microscopy (SEM) and transmission electron microscopy (TEM) images of nHA and vHA (scale bar: 5 μm for SEM and 200 nm for TEM). **e** Compressive stress-strain curves of different hydrogels measured using a universal testing machine (n=3). **f** Compressive Young's modulus of different hydrogels (n=3). **g** Rheological characterization of the scaffolds. Storage modulus (G′) and loss modulus (G″) were measured as a function of frequency from 0.1 to 10 Hz within the linear viscoelastic region (n=3). **h** Representative SEM images of the three Gel-based scaffolds (Gel, Gel-nHA, and Gel-vHA) (scale bar: 100 μm). **i** Quantitative analysis of swelling ratio in PBS (n=3). **j** Degradation curves in PBS showing mass remaining ratio (n=3). Data are represented as the mean ± SD (n=3). ns, no significance; $^{*}P < 0.05$; $^{***}P < 0.001$.

Supplementary Figure 10

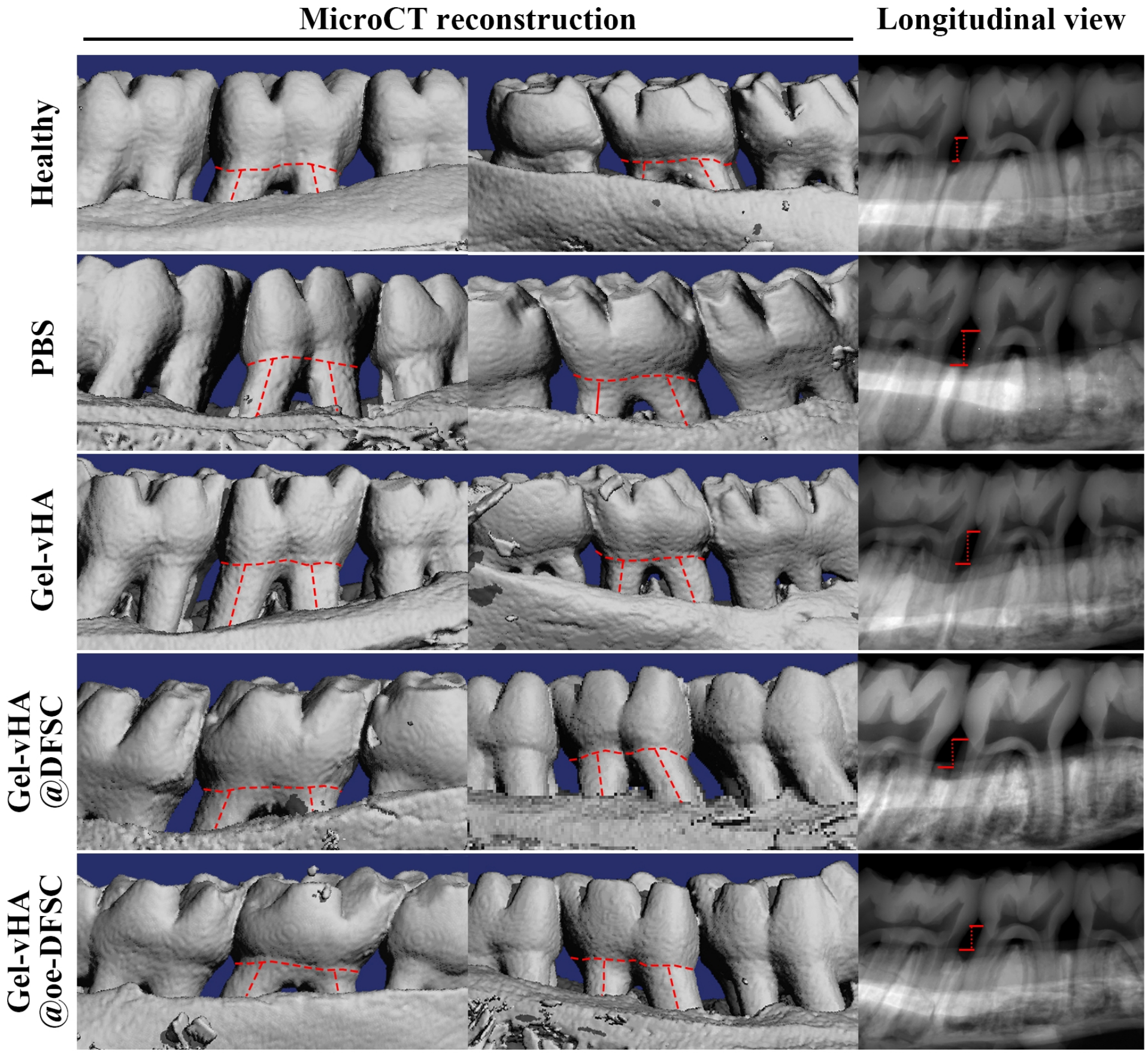

Supplementary Figure 10. Micro-CT analysis of alveolar bone regeneration in rat maxillary second molars at four weeks post-treatment. Representative three-dimensional (3D) reconstructed images (left and middle panels) and two-dimensional (2D) longitudinal cross-sections (right panels) are shown for the Healthy, PBS, Gel-vHA, Gel-vHA@DFSC, and Gel-vHA@oe-DFSC groups. Red dashed lines indicate the alveolar bone level at the mesial and distal roots.
